# Auditory paradigm-dependent EEG feature screening and representation for distinguishing consciousness states in male patients with disorders of consciousness

**DOI:** 10.3389/fneur.2026.1927313

**Published:** 2026-09-30

**Authors:** Fang Duan, Jianming Chen, Zheng Yan, Xiongping Cao, Guanyu Xiong, Fang Li

**Affiliations:** 1 College of Information Science and Engineering, Huaqiao University, Xiamen, China; 2 School of Information Engineering, Minnan University of Science and Technology, Quanzhou, China; 3 School of Biomedical Engineering, University of British Columbia, Vancouver, BC, Canada; 4 Parkinson Medical Center, Beijing Rehabilitation Hospital, Capital Medical University, Beijing, China

**Keywords:** auditory stimulation, consciousness state classification, disorders of consciousness, EEG, feature representation, nonlinear features

## Abstract

**Introduction:**

Accurate assessment of disorders of consciousness (DoC), including minimally conscious state (MCS) and unresponsive wakefulness syndrome (UWS/VS), remains a major challenge in clinical neurorehabilitation. EEG-based biomarkers offer a non-invasive approach for characterizing residual brain activity, yet their discriminative sensitivity may depend on auditory stimulus design.

**Methods:**

EEG recordings were obtained from 15 male patients with DoC during four auditory stimulation paradigms. Nonlinear, spectral power, and graph-theoretic features were extracted, yielding 136 candidate features. All individual features were independently evaluated using patient-level leave-one-out cross-validation (LOOCV) to characterize paradigm- and feature-domain-specific predictive performance. In a complementary exploratory optimization analysis, univariate statistical screening identified 26 features with nominal group differences (*p* < 0.05); these features were subsequently subjected to within-fold K-means clustering and principal component analysis (PCA), and compared with directly combined feature representations for MCS versus UWS/VS classification and CRS-R score regression.

**Results:**

In the single-feature benchmark, the reversed-word sequence paradigm showed relatively higher averaged classification performance, while nonlinear features demonstrated comparatively stronger classification characteristics than spectral power and graph-theoretic features. In the optimization analysis, the PCA-derived representation achieved competitive classification performance, whereas the direct combination of all statistically screened features achieved the highest observed classification accuracy. Regression performance was generally limited.

**Discussion:**

These findings suggest that auditory stimulus design and EEG feature representation may influence the discrimination of consciousness states in DoC. In this small-sample exploratory study, reversed-word auditory stimulation and nonlinear EEG features showed promising discriminative characteristics, while direct feature combination provided additional predictive information for categorical classification. Further validation in larger and independent cohorts is required.

## 1 Introduction

Disorders of consciousness (DoC) arise from severe impairments in the neural systems supporting arousal and awareness (1), typically manifesting as Vegetative State/Unresponsive Wakefulness Syndrome (UWS/VS) (2) and Minimally Conscious State (MCS) (3). These conditions reflect different levels of disruption in large-scale brain network integration. Accurate assessment of consciousness level remains a central challenge in neurocritical care and rehabilitation.

Currently, the Coma Recovery Scale-Revised (CRS-R) (4) is widely used as the clinical reference for DoC diagnosis. This behavioral scale evaluates auditory, visual, motor, and communication responses. However, its reliability is constrained by several factors, including motor impairments, fluctuating arousal levels, and subjective interpretation. Reported misdiagnosis rates can reach 37–43% (5–7), particularly due to the difficulty of detecting subtle but meaningful responses in MCS patients. These limitations highlight the need for objective neurophysiological measures to complement behavioral assessment. Importantly, EEG-based measures should be interpreted as correlates of CRS-R or related behavioral indices, rather than direct measurements of the underlying latent construct of consciousness.

Electroencephalography (EEG) provides a non-invasive, cost-effective, and bedside-accessible approach for probing brain function in DoC patients (8–10). In recent years, EEG’s contributions to the diagnosis and prognosis of DoC patients have become increasingly evident (11–13). Both resting-state and task-based EEG have demonstrated potential in differentiating consciousness levels and predicting clinical outcomes (14, 46, 47). In particular, task-related paradigms can enhance the detection of residual cognitive processing (15). For example, auditory stimulation paradigms have been shown to elicit differential neural responses between MCS and UWS/VS patients, reflecting preserved higher order processing in a subset of patients (16).

Within EEG feature analysis, nonlinear dynamical metrics are closely linked to theories proposing that consciousness depends on the complexity of brain activity (17, 18). Measures such as approximate entropy, sample entropy, and Lempel–Ziv complexity quantify the irregularity and diversity of neural signals, reflecting the brain’s capacity for information integration. Clinical studies have shown that reduced EEG complexity is associated with impaired consciousness and poorer prognosis, whereas higher complexity is observed in patients who retain or recover conscious awareness (14, 19, 20).

Spectral features, on the other hand, reflect frequency specific neural oscillations that are closely associated with the level of consciousness. Prior studies consistently report increased delta power and reduced alpha and theta activity in UWS/VS patients, while MCS patients exhibit relatively preserved higher-frequency oscillations (21, 22). Moreover, theta and alpha activity in temporoparietal regions has been shown to be higher in MCS compared to UWS/VS, with alpha power positively correlated and delta power negatively correlated with CRS-R scores (21, 23, 24).

Functional connectivity characterizes interactions among distributed brain regions and provides a network-level perspective on the neural basis of consciousness (25, 26). Within a graph-theoretical framework, these interactions can be quantified in terms of integration and segregation, which are closely related to cognitive function (27, 28). In DoC, impaired awareness is consistently associated with disrupted large-scale brain networks. EEG studies further show that functional connectivity is generally weaker in UWS/VS patients compared to MCS patients, particularly in long-range cortical networks, while partially preserved connectivity in MCS reflects residual capacity for information integration (29). These findings suggest that connectivity-based features provide complementary information for distinguishing levels of consciousness.

Despite advances in EEG-based assessment of disorders of consciousness, most existing studies rely on a single type of auditory stimulation paradigm, which limits the understanding of how stimulus structure influences residual neural processing. Auditory perception in DoC patients is not a uniform process but rather reflects varying degrees of preserved cortical and subcortical integration. Different auditory paradigms may therefore engage distinct levels of cognitive processing, ranging from basic sensory encoding to higher-order linguistic and semantic integration.

However, systematic comparisons of multiple auditory paradigms within a unified analytical framework remain limited. In particular, it is unclear whether more cognitively demanding auditory stimuli can enhance the detectability of residual consciousness-related EEG signatures. Addressing this gap may improve the robustness of EEG-based biomarkers and provide new insights into residual auditory cognition in DoC patients.

Therefore, the present study aims to systematically compare EEG responses across four auditory paradigms in patients with DoC. We investigate whether different stimulus structures influence the discriminative performance of EEG features and whether nonlinear, spectral, and graph-theoretic features provide complementary information for consciousness assessment. Specifically, the objectives of this study are to: (1) compare the discriminative performance of EEG feature types across auditory paradigms for distinguishing MCS and UWS/VS; (2) evaluate associations between EEG features and CRS-R scores; and (3) assess the performance of a low-dimensional representation relative to individual and combined feature sets.

## 2 Methods

### 2.1 Subjects

A total of 18 patients with DoC were recruited from the China Rehabilitation Research Center (Beijing Bo’ai Hospital). Patients were included if they met the following criteria: (1) a clinical diagnosis of DoC based on CRS-R assessment, and (2) the ability to undergo bedside EEG recording during auditory stimulation paradigms.

EEG recordings were acquired at the bedside for all participants. Three recordings were excluded due to auditory threshold abnormalities, fever during acquisition, and poor EEG signal quality, respectively. The final dataset included 15 patients. All patients were assessed using the CRS-R prior to EEG recording. Among them, 6 patients were diagnosed with MCS and 9 with UWS/VS. Clinical information including etiology (e.g., intracerebral hemorrhage and traumatic brain injury), surgical history, and medication use was collected from medical records. Additional patient information is provided in the Supplementary material. Table 1 summarizes the basic information of patients. All participants were male.

**Table 1:** Patient information.

No.	Age	Sex	Etiology	Time since injury	CRS-R	Diagnosis
1	50	Male	ICH	~5 months	7	UWS/VS
2	68	Male	ICH	~2–3 months	11	MCS
3	46	Male	TBI	~1 month	17	MCS
4	65	Male	ICH	~4 months	2	UWS/VS
5	53	Male	ICH	~1–2 months	3	UWS/VS
6	70	Male	ICH	~11 months	7	UWS/VS
7	53	Male	ICH	~2–3 months	10	MCS
8	51	Male	TBI	~7 months	7	UWS/VS
9	69	Male	ICH	~2–3 months	7	MCS
10	44	Male	ICH	~1 month	23	MCS
11	69	Male	TBI	~3 months	7	UWS/VS
12	64	Male	ICH	~3 months	7	UWS/VS
13	62	Male	ICH	~1 month	4	UWS/VS
14	41	Male	ICH	~10 months	2	UWS/VS
15	53	Male	TBI	~1 month	9	MCS

ICH, intracerebral hemorrhage; TBI, traumatic brain injury.

### 2.2 Experimental paradigms

Four auditory paradigms were designed and implemented using PsychoPy 2022: Pure-tone, Name-calling, Reversed-word sequence, and Different-word sequence paradigms. The experimental paradigm design is shown in Figure 1.

**Figure 1:**
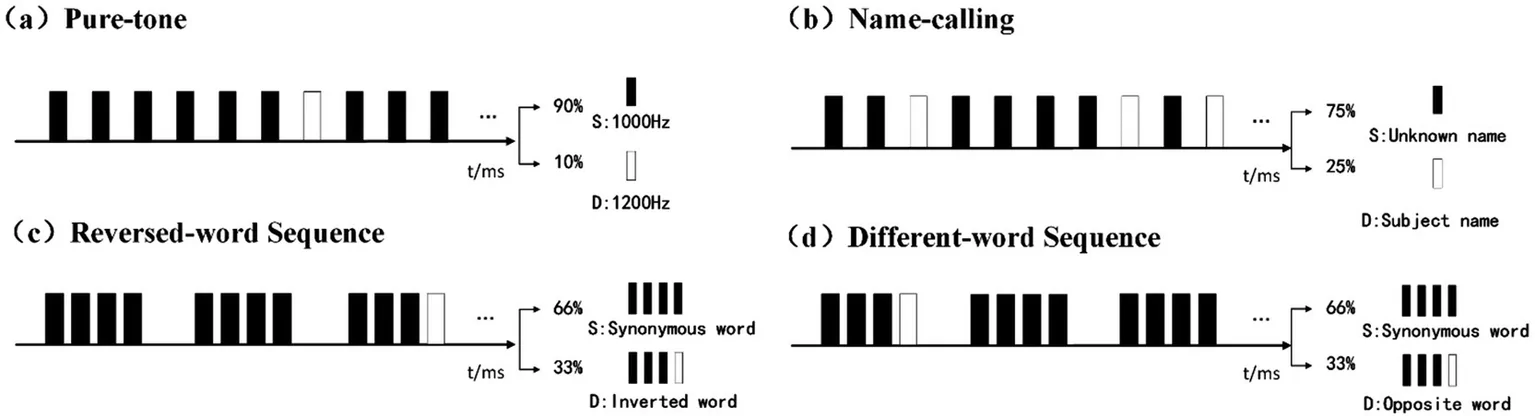
Paradigm design diagram. **(a)** Pure-tone paradigm. **(b)** Name-calling paradigm. **(c)** Reversed-word sequence paradigm. **(d)** Different-word sequence paradigm.

Pure-tone Paradigm: The standard stimulus was a 1,000 Hz sine tone, while the deviant stimulus was a 1,200 Hz sine tone. Both stimuli appear randomly at a 9:1 ratio. Each stimulus lasted 0.05 s, with random interstimulus intervals of 0.5–0.6 s. A total of 1,000 pure tone stimuli were presented.

Name-Calling Paradigm: Standard stimuli consist of strangers calling the participant’s name, lasting 0.5–0.6 s. Abnormal stimuli involve relatives calling the name, lasting ≤0.8 s. Both stimuli appear randomly at a 3:1 ratio. Interstimulus intervals are randomly distributed between 0.9–1.0 s. A total of 400 name-calling stimuli are presented.

Reversed-word Sequence Paradigm: Four words appear in groups. Standard stimuli consist of four synonyms. Deviant stimuli differ by reversing the audio of the fourth word. Both stimuli appeared randomly at a 2:1 ratio. Each word is played for 0.3–0.6 s, with random intervals of 0.4–0.5 s within each group. Intergroup intervals are 2 s, totaling 120 word sequence groups.

Different-word Sequence Paradigm: Four words appear in a group. The standard stimulus consists of four synonyms. The deviant stimulus differs from the standard stimulus in that the fourth word is an antonym. Both stimuli appeared randomly at a 2:1 ratio. All other aspects are consistent with the Reversed Word Sequence Paradigm.

These paradigms were designed to probe different levels of auditory and cognitive processing, ranging from basic sensory perception to higher-order linguistic integration. All four paradigms were conducted on the same day. The presentation order was fixed (Reversed-word sequence → Different- word sequence → Name-calling → Pure-tone) rather than randomized, to ensure procedural consistency across patients. Potential order effects cannot be fully excluded and are considered in the interpretation of the results.

### 2.3 Overview of the analytical framework

The analytical framework consisted of two complementary analysis streams. The research framework of the article is shown in Figure 2. The first stream was designed to characterize the predictive potential of individual EEG features. All candidate features extracted across the four auditory paradigms were individually evaluated within a patient-level leave-one-out cross-validation (LOOCV) framework using classification and regression models. The resulting single-feature predictions were subsequently summarized according to auditory paradigm and feature domain.

**Figure 2:**
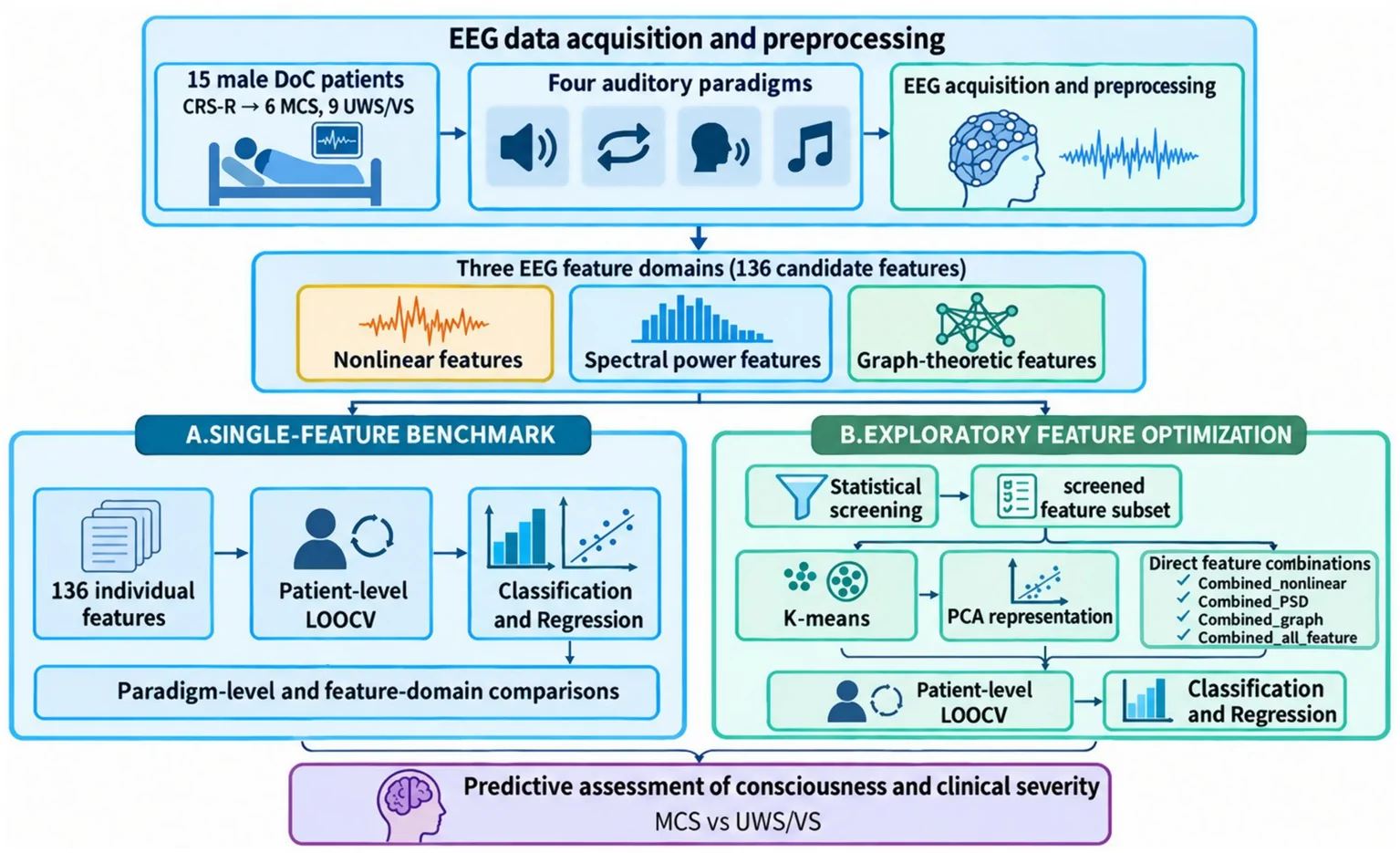
Research framework diagram.

The second stream was designed to evaluate an exploratory feature optimization and representation strategy. Candidate features were first screened using exploratory univariate statistical analyses, after which the retained feature subset was subjected to within-fold K-means-based redundancy reduction and principal component analysis (PCA) (30) within the LOOCV framework. In parallel, directly combined representations of the retained nonlinear, spectral power, and graph-theoretic features, as well as their overall combination, were evaluated.

Both analysis streams used the same patient-level LOOCV framework for predictive evaluation, with each held-out patient serving exclusively as the test subject in the corresponding iteration. The two streams therefore provided complementary information: the single-feature analysis characterized the intrinsic predictive patterns of the candidate EEG features, whereas the optimization stream examined whether statistical screening, redundancy reduction, and dimensionality reduction could provide a more compact informative representation.

### 2.4 EEG acquisition and preprocessing

EEG signals were recorded using the standard 19-channel montage according to the international 10–20 system, including Fp1, Fp2, F3, F4, F7, F8, Fz, T3, T4, T5, T6, C3, C4, Cz, P3, P4, Pz, O1, O2. Data acquisition employed the NeuSen NSM2 amplifier and EEGCap32- pony software platform at a sampling rate of 1,000 Hz. The reference electrode was the vertex electrode, with each electrode maintaining an impedance of less than 5kΩ relative to the scalp. Before the experiment, a qualified clinician assessed each patient’s clinical condition and suitability for participation in the EEG experiment. EEG recordings were conducted on site by trained EEG acquisition personnel, who remained present throughout the recording sessions to continuously monitor the EEG acquisition process and the participant’s condition. Preprocessing of EEG signals included electrode screening, removal of 50 Hz power line interference, 0.5–40 Hz bandpass filtering, interpolation of bad leads, ICA-based removal of ocular and muscular artifacts, whole brain average re- referencing, EEG segmentation, and segment level filtering.

ICA was performed on the continuous EEG recordings before stimulus-locked segmentation. A total of 19 independent components were obtained according to the number of EEG channels. Based on waveform characteristics and scalp distribution, components representing artifacts such as eye movements are identified and subsequently removed. No more than two components were removed from each recording to minimize distortion of neural signals. Regarding data segmentation, all time windows begin 100 milliseconds before the onset of the stimulus. The post stimulus window varies across different paradigms: 500 milliseconds for the pure-tone paradigm, and 1,000 milliseconds for the name-calling, reverse-word sequence, and different-word sequence paradigms. After segmentation, baseline correction is performed using the pre-stimulus time window. The inclusion of the baseline segment reflects ongoing brain activity and was treated consistently across conditions, allowing relative comparisons between groups. Regarding data filtering, both automated and manual screening were used to remove anomalous waveforms. The automated filtering criteria were as follows: if more than 1% of the data points in two or more channels exceeded the range of ±100 μV, or exceeded three standard deviations of the mean amplitude, that data segment was discarded. Across all paradigms, the proportion of discarded data segments did not exceed 10% of the total data segments for that paradigm, ensuring sufficient data retention for reliable analysis. Representative EEG visualizations for the four auditory paradigms are provided in the Supplementary material, including 19-channel stimulus-locked EEG waveforms, RMS-based scalp topographies, and PSD profiles.

### 2.5 Feature extraction

To comprehensively characterize EEG activity, three complementary feature domains were extracted from each stimulus-locked epoch: nonlinear features, spectral power features, and graph-theoretic features. Feature extraction was performed separately for each of the four auditory paradigms. For each paradigm, five nonlinear features, five spectral power features, and twenty-four graph-theoretic features were calculated, yielding 34 features per paradigm and 136 candidate features per participant across the four paradigms.

For nonlinear and spectral power features, channel-level measures were averaged across the 19 EEG channels to obtain whole-brain representations. For graph-theoretic features, network metrics were derived from wPLI-based connectivity matrices and averaged across nodes to obtain global network descriptors. For each subject and paradigm, feature values were first calculated for individual epochs and then averaged across epochs to obtain a subject-level feature representation. Each epoch included the pre-stimulus baseline (−100 ms) and the corresponding post-stimulus interval, and features were calculated from the complete epoch to maintain consistency across feature types.

#### 2.5.1 Nonlinear features

Nonlinear features were used to characterize the complexity, irregularity, and dynamical characteristics of EEG signals. Five nonlinear measures were extracted: approximate entropy (31), sample entropy, permutation entropy (32), spectral entropy (33), and Lempel–Ziv complexity (34). Approximate entropy and sample entropy were calculated using an embedding dimension of m = 2 and a tolerance of r = 0.2 × SD. Permutation entropy was calculated using the default embedding dimension and delay parameters. Spectral entropy was derived from the power spectral density using the EEG sampling frequency. For Lempel–Ziv complexity, the EEG signal was binarized according to the specified signal-based threshold and normalized complexity was subsequently calculated. All nonlinear features were first calculated separately for each EEG channel and then averaged across the 19 channels to obtain whole-brain feature representations.

#### 2.5.2 Spectral power features

Spectral power features were extracted to characterize the distribution of neural activity across frequency bands. Relative power was calculated for five canonical frequency bands: delta (1–4 Hz), theta (4–8 Hz), alpha (8–14 Hz), beta (14–30 Hz), and gamma (30–40 Hz). For each channel, the power within each band was normalized by total power to obtain relative measures. Similar to nonlinear features, these values were then averaged across all channels to yield whole brain spectral descriptors. All spectral features were computed over the entire epoch corresponding to each trial. No sliding- window segmentation or time–frequency decomposition was applied, ensuring that the extracted features represent global spectral characteristics of each epoch.

#### 2.5.3 Graph-theoretic features

Graph-theoretic features were derived from functional connectivity networks constructed using the weighted phase lag index (wPLI) (35), which quantifies phase synchronization between EEG signals while reducing the influence of volume conduction. The wPLI was calculated separately over six frequency ranges: delta, theta, alpha, beta, gamma, and full band. For each frequency range, a weighted connectivity matrix was constructed without applying an additional sparsity threshold. Four graph-theoretic metrics were then calculated from each weighted network: degree, clustering coefficient, closeness centrality, and betweenness centrality (36). These metrics characterize different aspects of network connectivity, local segregation, global communication efficiency, and nodal centrality, respectively. Each metric was averaged across network nodes to obtain a global graph-theoretic descriptor. Thus, 4 graph-theoretic metrics × 6 frequency ranges yielded 24 graph-theoretic features for each auditory paradigm.

### 2.6 Exploratory univariate statistical screening

To characterize group-level differences and define the candidate feature subset for the subsequent feature optimization analysis, an exploratory univariate statistical screening was performed for all candidate EEG features. For each feature, data normality was assessed using the Shapiro–Wilk test. Independent-samples *t*-tests were used for normally distributed features, whereas Mann–Whitney U tests were used for non-normally distributed features to compare MCS and UWS/VS patients.

Cohen’s d was calculated for each feature to quantify the magnitude of the between-group difference. Features meeting the nominal screening criterion of *p* < 0.05 were retained for the subsequent feature optimization strategy. No correction for multiple comparisons was applied because this analysis was intended as an exploratory screening procedure rather than confirmatory inference.

Importantly, this statistical screening was conducted at the full-cohort level before LOOCV and was used only to define the candidate feature subset for the optimization analysis. The single-feature benchmark analysis described in Section 2.8 used the full candidate feature set without applying this preliminary screening.

### 2.7 Exploratory feature optimization and PCA representation

To investigate whether a more compact and less redundant EEG feature representation could provide competitive predictive performance, the feature subset identified by the exploratory statistical screening was subjected to an additional optimization procedure. A patient-level LOOCV framework was used. In each iteration, one patient was held out as the test subject and the remaining 14 patients constituted the training set.

Let M denote the number of features retained after preliminary statistical screening. The training feature matrix in fold f was represented as

$$X_{\text{train}}^{\left(f\right)}\in R^{14\times M}$$

The corresponding held-out patient was represented as:

$$X_{\text{test}}^{\left(f\right)}\in R^{1\times M}$$

Within each training fold, K-means (37, 38) clustering was applied to reduce redundancy among the screened features. For feature j , its representation across the 14 training participants was defined as:

$$v_{j}^{\left(f\right)}={\left[x_{1j}^{\left(f\right)},x_{2j}^{\left(f\right)},\cdots ,x_{14j}^{\left(f\right)}\right]}^{T}\in R^{14},j=1,\cdots ,M$$

Thus, K-means clustering was performed on the feature vectors rather than on the participants. The number of clusters was determined using the elbow criterion. Within each cluster, the feature showing the strongest association with the training labels, quantified by Pearson correlation, was selected as the representative feature. If $K_{f}$ representative features were retained in fold f , the reduced training matrix was represented as:

$$X_{\mathrm{rep}}^{\left(f\right)}\in R^{14\times K_{f}}$$

The clustering structure and representative-feature selection were estimated using the training data only.

PCA was subsequently applied to the fold-specific representative feature matrix. After centering the training data, the PCA transformation was expressed as:

$$Z^{\left(f\right)}=X_{\mathrm{rep}}^{\left(f\right)}W^{\left(f\right)}$$

Where $X_{\mathrm{rep}}^{\left(f\right)}$ denotes the centered representative-feature matrix for the training data in fold f , and $W^{\left(f\right)}$ denotes the PCA loading matrix estimated from the training data in that fold. Components were retained until the cumulative explained variance reached at least 95%:

$$\frac{\sum_{r=1}^{q_{f}}\lambda_{r}^{\left(f\right)}}{\sum_{r=1}^{K_{f}}\lambda_{r}^{\left(f\right)}}\geq 0.95$$

Where $\lambda_{r}^{\left(f\right)}$ denotes the eigenvalue of the r -th principal component and $q_{f}$ denotes the number of retained principal components in fold f . The resulting PCA representation was therefore composed of the first $q_{f}$ principal components:

$$Z_{q_{f}}^{\left(f\right)}=[PC_{1}^{\left(f\right)},PC_{2}^{\left(f\right)},\cdots ,PC_{q_{f}}^{\left(f\right)}]$$

More generally, the n -th principal component was defined as:

$$PC_{n}^{\left(f\right)}=X_{c}^{\left(f\right)}w_{n}^{\left(f\right)},n=1,\cdots ,q_{f}$$

Where $w_{n}^{\left(f\right)}$ is the loading vector corresponding to the n -th retained principal component.

The K-means-based feature reduction and PCA transformation were fitted exclusively using the training data in each LOOCV iteration. The resulting transformations were then applied unchanged to the held-out patient to obtain its corresponding reduced and PCA-derived representations. The initial statistical screening was performed before LOOCV and was therefore not repeated within individual folds. Accordingly, the optimization analysis was treated as exploratory, and its performance estimates were interpreted as internal exploratory estimates rather than independently validated measures of generalizability.

### 2.8 Classification and regression analysis

Two complementary predictive evaluation streams were considered. First, all candidate EEG features were evaluated individually within the patient-level LOOCV framework to characterize their intrinsic classification and regression potential. These single-feature results were subsequently summarized at the auditory-paradigm and feature-domain levels. Second, the statistically screened feature subset was evaluated using the exploratory optimization strategy described in Section 2.7. The resulting PCA-derived representation was compared with directly concatenated feature representations. These concatenated representations were generated by merging statistically pre-screened features grouped by feature type: combined_nonlinear for combined nonlinear features, combined_PSD for combined power-spectral-density features, combined_graph for combined graph-theoretic features, and combined_all_feature for the combination of all pre-screened features.

For classification, five conventional machine-learning models were evaluated: logistic regression (LR) (39), support vector machine with a linear kernel (SVM) (40), random forest (RF) (41), k-nearest neighbors (KNN) (39), and decision tree (DT) (42). These models were used as a common predictive evaluation framework rather than as methodological innovations. Models were fitted using the training data in each LOOCV iteration and subsequently applied to the held-out patient. Classification performance was assessed using accuracy, precision, recall, Cohen’s kappa, and the area under the receiver operating characteristic curve (AUC), with MCS defined as the positive class.

For regression, the relationship between EEG representations and continuous CRS-R scores was evaluated using ordinary least squares (OLS) linear regression and support vector regression (SVR) (43). The SVR model was implemented using scikit-learn with default hyperparameters, including C = 1.0, ϵ = 0.1, and γ = scale. Regression performance was evaluated using the coefficient of determination (*R*^2^). Statistical significance was additionally reported for applicable regression analyses according to the corresponding model and statistical procedure.

For the single-feature benchmark, predictions were generated independently for each candidate feature and aggregated across the 15 LOOCV iterations before calculating the corresponding performance metrics. For the optimization analysis, predictions were generated separately for each optimized or combined representation and similarly aggregated across the 15 held-out patients. Performance comparisons across paradigms, feature domains, and optimized representations were interpreted as exploratory, given the small sample size, class imbalance, and the preliminary statistical screening used in the optimization route. To enhance reproducibility, feature data and corresponding processing code are provided.

## 3 Results

### 3.1 Exploratory statistical screening of EEG features

Group-level statistical comparisons between MCS and UWS/VS patients were performed for all 136 candidate EEG features extracted across the four auditory paradigms. Depending on data normality, either independent two-sample *t*-tests or Mann–Whitney U tests were applied. Cohen’s d was additionally calculated for each feature to quantify the magnitude of between-group differences.

As summarized in Figure 3a, 26 features met the nominal screening criterion of *p* < 0.05, including 14 nonlinear features, 9 spectral power features, and 3 graph-theoretic features. These corresponded to 70.0% of the nonlinear features, 45.0% of the spectral power features, and 3.1% of the graph-theoretic features, respectively. Thus, nonlinear features showed the highest proportion of nominally significant features, followed by spectral power features, whereas relatively few graph-theoretic features met the screening criterion.

**Figure 3:**
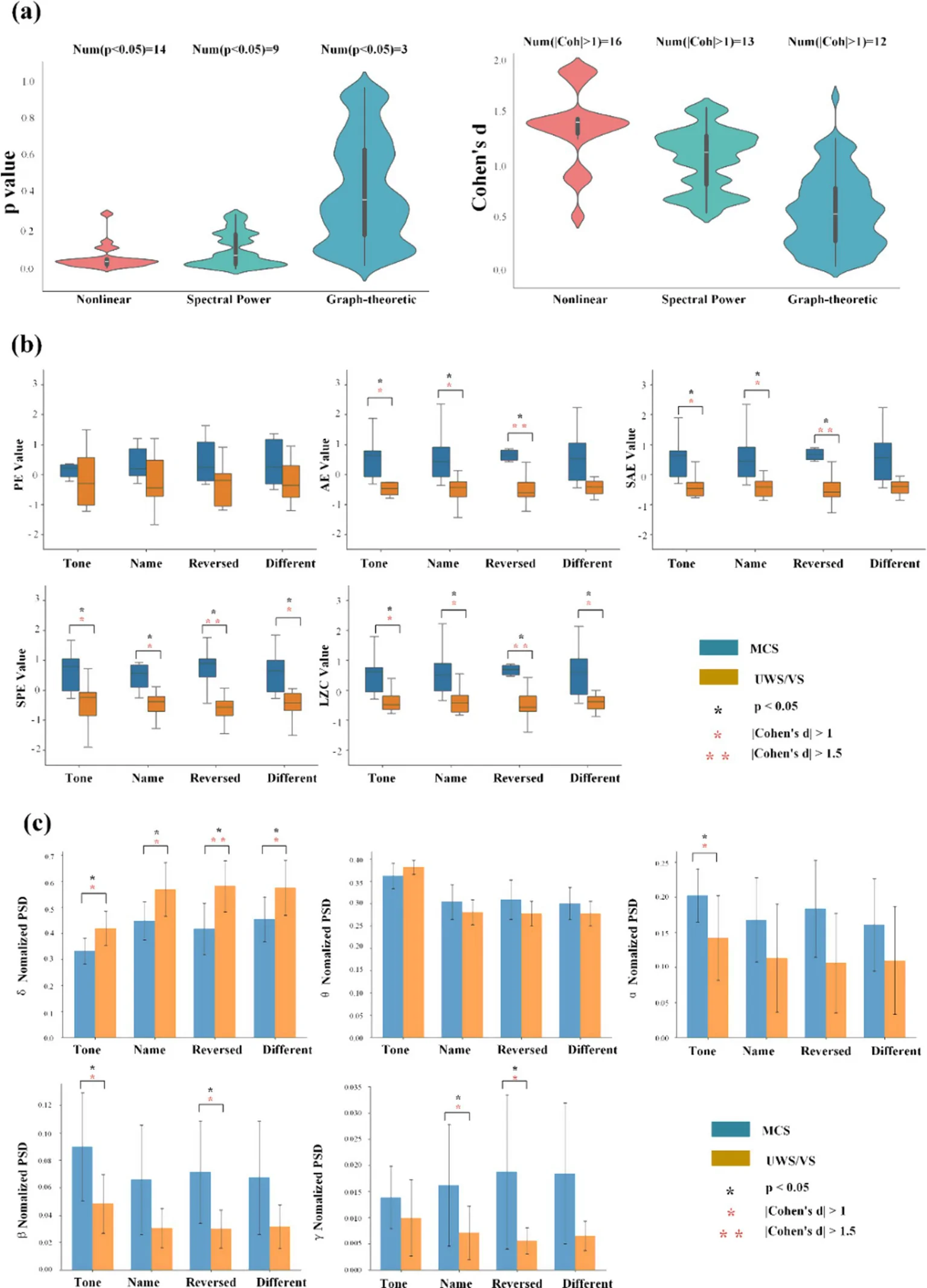
Feature statistics results. **(a)** *p*-value and Cohen’s d-value distribution maps. **(b)** Nonlinear feature box plot. **(c)** Power spectrum characteristic box diagram.

In terms of effect size, 16 nonlinear features, 13 spectral power features, and 12 graph-theoretic features exhibited large effects (|d| > 1). Because 136 univariate comparisons were performed without correction for multiple comparisons, these findings were considered exploratory and hypothesis-generating rather than confirmatory evidence of group differences or independently validated biomarkers. Detailed statistics for all candidate features, including the statistical test, *p*-value, and Cohen’s d, are provided in the Supplementary material.

As illustrated in Figure 3b, at the individual feature level, several nonlinear measures showed relatively consistent group differences across auditory paradigms. Approximate entropy, sample entropy, spectral entropy, and Lempel–Ziv complexity generally exhibited higher values in MCS patients than in UWS/VS patients, whereas permutation entropy showed less consistent group differences. For spectral power features (Figure 3c), UWS/VS patients generally exhibited higher relative delta power, whereas MCS patients showed relatively higher power in faster frequency bands, particularly beta and gamma. Differences in theta and alpha power were less consistent across paradigms.

When stratified by auditory paradigm, the reversed-word sequence paradigm showed relatively larger effect sizes for several nonlinear and spectral power features than the other paradigms. This pattern suggests greater group-level separability under the reversed-word condition in the present cohort. However, given the exploratory nature of the analysis, the small sample size, and the absence of multiple-comparison correction, this finding should not be interpreted as definitive evidence of paradigm superiority. The 26 statistically screened features identified in this analysis were subsequently used for the exploratory feature optimization strategy, whereas all 136 individual features were retained for the independent single-feature predictive benchmark analysis.

### 3.2 Paradigm-level comparison of single-feature performance

To characterize the influence of auditory paradigm design on the predictive potential of individual EEG features, all 136 candidate features were independently evaluated within the patient-level LOOCV framework using the classification and regression models described above. For paradigm-level comparison, single-feature results were grouped according to the four auditory paradigms: Pure-tone, Name-calling, Reversed-word sequence, and Different-word sequence. Performance metrics were averaged across the individual features and classifiers within each paradigm to obtain an overall descriptive estimate of single-feature performance.

It should be noted that such aggregated averages combine results from multiple feature–classifier combinations, including potentially unstable or non-informative models. Therefore, these averaged metrics are presented as exploratory summaries rather than as definitive indicators of paradigm-specific performance. Notably, due to the use of aggregated averages, only the coefficient of determination is presented in the regression result summaries.

At the classification level, the reversed-word sequence paradigm showed relatively higher averaged performance compared with the other paradigms (Table 2). It achieved the highest values across all major classification metrics, including accuracy (0.611), precision (0.334), recall (0.313), Cohen’s kappa (0.123), and AUC (0.403). This pattern suggests that the reversed-word sequence paradigm may provide a relatively more favorable condition for distinguishing between MCS and UWS/VS patients within the current experimental setting.

**Table 2:** Summary of single-feature classification and regression performance across auditory paradigms and EEG feature domains.

Dimension	Name	Classification					Regression (CRS-R)	
		Accuracy	Precision	Recall	Kappa	AUC	OLS(*R*^2^)	SVR(*R*^2^)
Paradigm	Tone	0.582	0.298	0.289	0.065	0.356	0.148	0.088
	Name	0.588	0.306	0.255	0.065	0.355	0.098	0.128
	Reversed	0.611	0.334	0.313	0.123	0.403	0.082	0.193
	Different	0.575	0.267	0.267	0.042	0.342	0.085	0.185
Feature	Nonlinear	0.635	0.367	0.343	0.172	0.412	0.104	0.216
	Spectral	0.603	0.297	0.280	0.099	0.352	0.046	0.135
	Graph	0.529	0.240	0.220	−0.050	0.329	0.159	0.094

At the regression level, where performance was evaluated based on prediction of continuous CRS- R scores, differences across paradigms were modest and less consistent. The reversed-word sequence paradigm achieved the highest *R*^2^ in SVR-based regression (*R*^2^ = 0.193), whereas OLS regression results showed small variations across paradigms, with the pure-tone paradigm yielding a slightly higher *R*^2^ (0.148). Overall, no paradigm demonstrated consistently superior performance across regression models.

Taken together, these findings suggest that the reversed-word sequence paradigm exhibits relatively higher exploratory performance in classification tasks, while its advantage is less pronounced in regression analyses. This pattern indicates that paradigm design may have a stronger influence on categorical discrimination than on continuous clinical estimation under the present framework. These observations are based on aggregated results and should be interpreted as descriptive trends rather than definitive comparative conclusions. Full classification and regression results for all individual features and classifiers are provided in the Supplementary material.

### 3.3 Feature type level performance

To characterize the predictive potential of different EEG feature domains, all 136 candidate features were independently evaluated at the single-feature level within the LOOCV framework. Results were grouped into three domains: nonlinear, spectral power, and graph-theoretic features. For each domain, performance metrics were first averaged across classifiers for each individual feature and then across features within the domain, yielding an overall descriptive estimate of domain-level performance.

As with the paradigm-level analysis, these aggregated metrics reflect averaged behavior across multiple feature–model combinations and may be influenced by variability in feature stability, model performance, and the number of features within each category. Therefore, these results are intended to provide an overall descriptive comparison rather than a statistically rigorous ranking of feature domains. Similarly, only the coefficient of determination is presented in the regression results due to the aggregated nature of these metrics.

At the classification level, nonlinear dynamical features showed relatively higher performance compared with spectral and graph-theoretic features (Table 2). Specifically, nonlinear features yielded moderately higher values in accuracy (0.635), precision (0.367), recall (0.343), Cohen’s kappa (0.172), and AUC (0.412). Spectral features demonstrated intermediate performance (e.g., accuracy = 0.603, kappa = 0.099), while graph-theoretic features showed comparatively lower classification performance and negative kappa values (−0.050).

At the regression level, based on prediction of continuous CRS-R scores, nonlinear features also showed relatively higher performance in SVR modeling (*R*^2^ = 0.216), compared with spectral (0.135) and graph-theoretic features (0.094). In contrast, OLS regression exhibited a different pattern, with graph-theoretic features yielding a relatively higher *R*^2^ (0.159), suggesting that different feature types may capture complementary aspects of clinical variability depending on the modeling approach.

Overall, nonlinear features demonstrated comparatively stronger exploratory classification characteristics at the single-feature level, whereas spectral power and graph-theoretic features provided potentially complementary information in specific regression settings. These findings should be interpreted cautiously because they were derived from aggregated single-feature performance in a small and imbalanced cohort. Full classification and regression results for all individual features and classifiers are reported in Supplementary material.

### 3.4 Evaluation of the exploratory feature optimization and representation strategy

To further evaluate the exploratory feature optimization strategy, the predictive performance of the statistically screened feature set was examined using both individual and integrated representations. The screened features were first considered individually within the optimization analysis, and the best-performing feature from each feature domain was selected as a representative baseline. These included spectral entropy under the reversed-word paradigm (SPE_R) for the nonlinear domain, gamma-band power under the reversed-word paradigm (PSD_Gamma_R) for the spectral power domain, and theta-band wPLI-based closeness centrality under the pure-tone paradigm (WPLI_Theta_Clo_T) for the graph-theoretic domain. These representative single features were then compared with the PCA-derived representation (PC1) obtained from K-means-based feature redundancy reduction and with the directly combined representation of all statistically screened features (Combined_all_feature).

In classification, PC1 achieved an accuracy of 0.773, precision of 0.747, recall of 0.667, Cohen’s kappa of 0.521, and AUC of 0.793. The results are shown in Table 3. Compared with the best-performing individual features within the statistically screened feature set, PC1 showed the strongest overall classification profile across the evaluated metrics. In particular, its AUC exceeded those of SPE_R (0.726), PSD_Gamma_R (0.572), and WPLI_Theta_Clo_T (0.750), while also achieving higher accuracy and Cohen’s kappa than the three representative individual features. These findings suggest that combining information from multiple screened features through PCA can provide a compact representation with strong discriminative performance.

**Table 3:** Classification performance of representative screened features and optimized representations.

Feature	Ave_ Accuracy	Ave_ Precision	Ave_ Recall	Ave_ Kappa	Ave_ AUC
PC1	0.773	0.747	0.667	0.521	0.793
SPE_R	0.747	0.720	0.600	0.455	0.726
PSD_Gamma_R	0.707	0.470	0.367	0.311	0.572
WPLI_Theta_Clo_T	0.760	0.655	0.833	0.520	0.750
Combined_all_feature	0.800	0.746	0.767	0.585	0.756

The directly combined representation, Combined_all_feature, achieved the highest observed classification accuracy (0.800) and Cohen’s kappa (0.585), with an AUC of 0.756. Thus, Combined_all_feature achieved higher peak accuracy than PC1, whereas PC1 provided a higher AUC. This pattern indicates a trade-off between retaining the full multivariate feature information and constructing a compact low-dimensional representation.

The regression results representing the characteristics of each category are shown in Table 4. In regression analyses focusing on continuous CRS-R scores, PC1 demonstrated limited predictive performance (OLS: *R*^2^ = 0.118, *p* = 0.211; SVR: *R*^2^ = 0.216, *p* = 0.081) and did not outperform the best-performing individual features (e.g., gamma power under the reversed paradigm, SVR *R*^2^ = 0.429, *p* = 0.008). This observation is consistent with the fundamental property of PCA, which identifies directions of maximal variance in the data without considering the target variable. As a result, the principal components may not align with the directions that are most predictive of CRS-R scores. In contrast, specific features such as band-limited spectral power may exhibit stronger direct associations with clinical scores, thereby achieving higher regression performance. Furthermore, CRS-R scores represent a coarse and potentially nonlinear clinical scale, which may depend on localized or feature-specific neural signatures rather than global variance patterns. Consequently, while PC1 captures dominant global structure in the feature space, it may attenuate subtle but clinically relevant variations required for accurate continuous prediction.

**Table 4:** Regression performance of representative screened features and optimized representations.

Feature	OLS		SVR	
	*R*^2^_score	p_ score	*R*^2^_ score	p_ score
PC1	0.118	0.211	0.216	0.081
SPE_R	0.175	0.121	0.187	0.107
PSD_Gamma_R	0.037	0.493	0.429	0.008
WPLI_Theta_Clo_T	0.020	0.618	0.001	0.924
Combined_all_feature	0.002	0.876	0.408	0.010

SPE, spectral entropy; R, flipped paradigm; T, pure tone paradigm; Clo, approaching centrality; Combined_all_feature, all feature combinations selected through statistical testing.

Overall, the optimization analysis indicates that PC1 provided a compact and competitive representation relative to the best individual features within the screened feature set, whereas direct combination of all screened features yielded the highest observed accuracy. These findings suggest complementary trade-offs between dimensional compactness and preservation of multivariate information, rather than a universally superior representation. Detailed results for all fusion and combined feature configurations are provided in Supplementary material.

## 4 Discussion

This study examined the influence of auditory paradigm design and EEG feature representation on the discrimination of MCS and UWS/VS using two complementary analytical streams: a single-feature benchmark and an exploratory feature optimization strategy. Three main findings emerged. First, the reversed-word sequence paradigm showed relatively higher classification performance at the single-feature level. Second, nonlinear features demonstrated comparatively stronger and more consistent classification characteristics than spectral power and graph-theoretic features. Third, the optimization analysis showed that PCA provided a compact representation with competitive classification performance, whereas direct combination of the screened features achieved higher peak accuracy. These findings suggest that both stimulus design and feature representation may influence EEG-based discrimination of consciousness states.

### 4.1 Comparison with existing approaches

To further contextualize the proposed framework, three representative EEG-based approaches were implemented and evaluated using the same patient-level leave-one-out cross-validation (LOOCV) framework (Table 5). These included (1) a conventional ERP-based approach using the amplitude and latency of the P3a component at the Cz electrode with an SVM classifier, following previously reported ERP-based approaches for the assessment of consciousness states (44); (2) an EEGNet-based deep-learning approach using raw EEG epochs (45), with epoch-level predictions averaged to obtain the patient-level prediction; and (3) a DOC-Forest-inspired Extra-Trees classifier using the 136 EEG features extracted in the present study (32).

**Table 5:** Comparison of representative EEG-based approaches for MCS versus UWS/VS classification.

Approach	Classification strategy	Accuracy	Precision	Recall	Kappa	AUC
ERP-based (44)	P3a-Cz + SVM	0.467	0	0	−0.25	0.611
EEGNet (45)	Raw EEG + EEGNet	0.533	0.429	0.500	0.054	0.426
DOC-Forest-inspired (32)	136 EEG features + Extra-Trees	0.733	0.750	0.500	0.412	0.667
Feature-optimized	Screening + K-means + PCA	0.773	0.747	0.667	0.521	0.793

As shown in Table 5, the ERP-based approach and EEGNet showed limited classification performance, with accuracies of 0.467 and 0.533 and AUCs of 0.611 and 0.426, respectively. In contrast, the DOC-Forest-inspired Extra-Trees approach achieved higher performance (accuracy = 0.733, AUC = 0.667), suggesting that the multidomain handcrafted EEG features may contain discriminative information for distinguishing MCS from UWS/VS. The proposed feature-representation optimization framework achieved the most favorable performance in the present cohort, with an accuracy of 0.773 and AUC of 0.793, together with a Kappa of 0.521, precision of 0.747, and recall of 0.667. These results suggest that reducing feature redundancy and constructing a compact representation may provide additional benefit beyond directly applying an ensemble classifier to the complete feature set. Nevertheless, given the small sample size, these findings should be considered preliminary and require validation in larger independent cohorts.

### 4.2 Effects of auditory paradigm design on consciousness-state discrimination

The reversed-word sequence paradigm showed the highest averaged classification performance among the four auditory conditions, although the absolute performance remained modest. This pattern was also broadly consistent with the group-level statistical results, in which several nonlinear and spectral power features showed relatively larger effect sizes under the reversed-word condition. The reversed-word stimulus may provide a stronger perturbation of expected auditory or linguistic processing, thereby eliciting neural responses that are more distinguishable between MCS and UWS/VS patients. However, this interpretation remains speculative because the present study did not directly isolate the neural effects of stimulus novelty or deviance.

Notably, the advantage of the reversed-word paradigm was more evident in classification than in CRS-R regression. This suggests that auditory paradigm design may contribute more strongly to categorical separation of consciousness states than to continuous estimation of clinical severity. Given the small sample size and modest overall performance, the reversed-word effect should be regarded as an exploratory finding requiring independent validation.

### 4.3 Discriminative characteristics of EEG feature domains

Nonlinear features showed the strongest overall classification profile in the single-feature benchmark, with higher averaged performance than spectral power and graph-theoretic features. This pattern was consistent with the statistical screening results, in which a larger proportion of nonlinear features showed nominal group differences. The relatively stronger performance of nonlinear measures may reflect their ability to characterize EEG complexity and dynamical organization, which may be particularly relevant to the altered brain dynamics associated with disorders of consciousness.

Nevertheless, no feature domain was uniformly optimal across all analyses. Spectral power and graph-theoretic features showed useful information in specific settings, particularly in some regression analyses. These findings suggest that different EEG feature domains may capture complementary aspects of residual brain activity rather than representing interchangeable measures of consciousness.

### 4.4 Feature optimization and representation

The exploratory optimization analysis further examined whether statistically screened features could be transformed into more compact predictive representations. K-means-based redundancy reduction followed by PCA produced a low-dimensional representation, with PC1 achieving competitive classification performance while substantially reducing feature dimensionality. This indicates that a considerable portion of the discriminative information may be represented in a lower-dimensional feature space.

Compared with the best-performing individual features within the screened feature set, PC1 provided a more compact representation with competitive classification performance. Direct combination of the screened features provided a complementary result. Combined_all_feature achieved the highest observed classification accuracy, whereas PC1 showed a higher AUC. This suggests a trade-off between compactness and retention of multivariate information: PCA provides a concise representation, whereas direct feature combination preserves more detailed feature-specific information. Importantly, these findings do not establish a universally superior representation, but rather indicate that the optimal representation may depend on the predictive objective.

### 4.5 Methodological considerations in performance interpretation

The classification and regression results should be interpreted differently. Classification performance was generally more informative than continuous CRS-R prediction, suggesting that the extracted EEG features may better capture broad differences between MCS and UWS/VS than the finer-grained variability represented by CRS-R scores.

The classification results were also sensitive to class imbalance and classifier behavior. With 9 UWS/VS and 6 MCS patients, some models favored the majority class, resulting in moderate accuracy but low precision, recall, kappa, or AUC. Therefore, accuracy alone may overestimate predictive utility, and complementary metrics are necessary for interpretation.

An additional methodological consideration concerns the exploratory optimization analysis. The initial statistical screening was performed before LOOCV, whereas K-means feature reduction and PCA were performed within each training fold. Consequently, the optimization results should be considered exploratory internal estimates and may be subject to optimistic bias. Larger studies should apply fully nested cross-validation to obtain more rigorous estimates of generalizability.

### 4.6 Limitations

Several limitations should be considered. First, the sample size was small (*n* = 15) and class distribution was imbalanced, limiting the stability and generalizability of the predictive estimates. Second, no healthy control group was included, limiting comparison with normative EEG characteristics. Third, no independent external validation cohort was available. Fourth, the initial statistical screening in the optimization analysis was performed before LOOCV, which may introduce optimistic bias. Fifth, the whole-brain feature representation reduced channel-specific information and therefore limited electrode-level interpretation. Sixth, the study focused on nonlinear, spectral power, and graph-theoretic features and did not examine other potentially informative representations, such as event-related potentials or stimulus-specific responses. Furthermore, the present analysis did not distinguish between standard and deviant stimuli within the auditory paradigms, primarily due to the small sample size, significant imbalance between the two trial types, and misalignment with the study’s focus on paradigm-level and feature-level neural effects.

## 5 Conclusion

This study investigated the influence of auditory paradigm design and EEG feature representation on the discrimination of consciousness states in patients with disorders of consciousness using complementary single-feature and feature-optimization analyses. In the single-feature benchmark, the reversed-word sequence paradigm showed relatively higher exploratory classification performance across the four auditory conditions, while nonlinear features demonstrated comparatively stronger and more consistent classification characteristics than spectral power and graph-theoretic features. The subsequent feature-optimization analysis showed that K-means-based redundancy reduction and PCA could provide a compact representation with competitive classification performance, whereas direct combination of the statistically screened features achieved the highest observed classification accuracy.

Overall, the findings suggest that both auditory stimulus design and EEG feature representation may contribute to the differentiation of MCS and UWS/VS. In particular, reversed-word stimulation and nonlinear EEG features showed promising exploratory discriminatory characteristics, while different feature representations provided complementary trade-offs between predictive performance and dimensional compactness. Given the small sample size, class imbalance, preliminary statistical screening before LOOCV, and lack of external validation, these findings should be considered hypothesis-generating. Larger, multicenter, and independently validated datasets are required to determine their robustness and clinical generalizability.

## Data Availability

The original contributions presented in the study are included in the article/Supplementary material, further inquiries can be directed to the corresponding authors.

## References

[1] Giacino JT Fins JJ Laureys S Schiff ND. Disorders of consciousness after acquired brain injury: the state of the science. Nat Rev Neurol. (2014) 10:99–114. doi: 10.1038/nrneurol.2013.279 24468878

[2] Laureys S Celesia GG Cohadon F Lavrijsen J León-Carrión J Sannita WG . Unresponsive wakefulness syndrome: a new name for the vegetative state or apallic syndrome. BMC Med. (2010) 8:68. doi: 10.1186/1741-7015-8-68, 21040571 PMC2987895

[3] Giacino JT Ashwal S Childs N Cranford R Jennett B Katz DI . The minimally conscious state. Neurology. (2002) 58:349–53. doi: 10.1212/WNL.58.3.349 11839831

[4] Giacino JT Kalmar K Whyte J. The JFK coma recovery scale-revised: measurement characteristics and diagnostic utility11No commercial party having a direct financial interest in the results of the research supporting this article has or will confer a benefit upon the authors or upon any organization with which the authors are associated. Arch Phys Med Rehabil. (2004) 85:2020–9. doi: 10.1016/j.apmr.2004.02.033 15605342

[5] Schnakers C Vanhaudenhuyse A Giacino J Ventura M Boly M Majerus S . Diagnostic accuracy of the vegetative and minimally conscious state: clinical consensus versus standardized neurobehavioral assessment. BMC Neurol. (2009) 9:35. doi: 10.1186/1471-2377-9-35, 19622138 PMC2718857

[6] Stender J Gosseries O Bruno MA Charland-Verville V Vanhaudenhuyse A Demertzi A . Diagnostic precision of PET imaging and functional MRI in disorders of consciousness: a clinical validation study. Lancet. (2014) 384:514–22. doi: 10.1016/s0140-6736(14)60042-8, 24746174

[7] Wang J Hu X Hu Z Sun Z Laureys S Di H. The misdiagnosis of prolonged disorders of consciousness by a clinical consensus compared with repeated coma-recovery scale-revised assessment. BMC Neurol. (2020) 20:343. doi: 10.1186/s12883-020-01924-9, 32919461 PMC7488705

[8] Alnagger N Cardone P Martial C Laureys S Annen J Gosseries O. The current and future contribution of neuroimaging to the understanding of disorders of consciousness. Presse Med. (2023) 52:104163. doi: 10.1016/j.lpm.2022.104163, 36796250

[9] Comanducci A Boly M Claassen J De Lucia M Gibson RM Juan E . Clinical and advanced neurophysiology in the prognostic and diagnostic evaluation of disorders of consciousness: review of an IFCN-endorsed expert group. Clin Neurophysiol. (2020) 131:2736–65. doi: 10.1016/j.clinph.2020.07.015, 32917521

[10] Scarpino M Lolli F Lanzo G Grippo A. What is the role of post acute EEG in prediction of late neurological outcome in severe disorders of consciousness? Future Neurol. (2020) 15:FNL36. doi: 10.2217/fnl-2019-0017

[11] Ballanti S Campagnini S Liuzzi P Hakiki B Scarpino M Macchi C . EEG-based methods for recovery prognosis of patients with disorders of consciousness: a systematic review. Clin Neurophysiol. (2022) 144:98–114. doi: 10.1016/j.clinph.2022.09.017, 36335795

[12] Sondag L Ruijter BJ Tjepkema-Cloostermans MC Beishuizen A Bosch FH van Til JA . Early EEG for outcome prediction of postanoxic coma: prospective cohort study with cost-minimization analysis. Crit Care. (2017) 21:111. doi: 10.1186/s13054-017-1693-2, 28506244 PMC5433242

[13] Toppi J Quattrociocchi I Riccio A D'Ippolito M Aloisi M Colamarino E . EEG-derived markers to improve prognostic evaluation of disorders of consciousness. IEEE J Biomed Health Inform. (2024) 28:6674–84. doi: 10.1109/jbhi.2024.3445118, 39150811

[14] Bai Y Lin Y Ziemann U. Managing disorders of consciousness: the role of electroencephalography. J Neurol. (2021) 268:4033–65. doi: 10.1007/s00415-020-10095-z, 32915309 PMC8505374

[15] Lancioni GE Singh NN O’Reilly MF Sigafoos J Desideri L. Music stimulation for people with disorders of consciousness: a scoping review. Brain Sci. (2021) 11:858. doi: 10.3390/brainsci11070858, 34203250 PMC8301821

[16] Qu S Wu X Tang Y Zhang Q Huang L Cui B . Analyzing brain-activation responses to auditory stimuli improves the diagnosis of a disorder of consciousness by non-linear dynamic analysis of the EEG. Sci Rep. (2024) 14:17446. doi: 10.1038/s41598-024-67825-w, 39075138 PMC11286939

[17] Demertzi A Tagliazucchi E Dehaene S Deco G Barttfeld P Raimondo F . Human consciousness is supported by dynamic complex patterns of brain signal coordination. Sci Adv. (2019) 5:eaat7603. doi: 10.1126/sciadv.aat7603, 30775433 PMC6365115

[18] Tononi G Boly M Massimini M Koch C. Integrated information theory: from consciousness to its physical substrate. Nat Rev Neurosci. (2016) 17:450–61. doi: 10.1038/nrn.2016.44, 27225071

[19] Altıntop ÇG Latifoğlu F Akın AK Bayram A Çiftçi M. Classification of depth of coma using complexity measures and nonlinear features of electroencephalogram signals. Int J Neural Syst. (2022) 32:2250018. doi: 10.1142/s0129065722500186 35300584

[20] Sarà M Pistoia F Pasqualetti P Sebastiano F Onorati P Rossini PM. Functional isolation within the cerebral cortex in the vegetative state: a nonlinear method to predict clinical outcomes. Neurorehabil Neural Repair. (2011) 25:35–42. doi: 10.1177/1545968310378508 20952634

[21] Lechinger J Bothe K Pichler G Michitsch G Donis J Klimesch W . CRS-R score in disorders of consciousness is strongly related to spectral EEG at rest. J Neurol. (2013) 260:2348–56. doi: 10.1007/s00415-013-6982-3, 23765089

[22] Rossi Sebastiano D Panzica F Visani E Rotondi F Scaioli V Leonardi M . Significance of multiple neurophysiological measures in patients with chronic disorders of consciousness. Clin Neurophysiol. (2015) 126:558–64. doi: 10.1016/j.clinph.2014.07.004, 25082091

[23] Fingelkurts AA Fingelkurts AA Bagnato S Boccagni C Galardi G. EEG oscillatory states as neuro-phenomenology of consciousness as revealed from patients in vegetative and minimally conscious states. Conscious Cogn. (2012) 21:149–69. doi: 10.1016/j.concog.2011.10.004, 22054641

[24] Marinazzo D Gosseries O Boly M Ledoux D Rosanova M Massimini M . Directed information transfer in scalp electroencephalographic recordings: insights on disorders of consciousness. Clin EEG Neurosci. (2014) 45:33–9. doi: 10.1177/1550059413510703 24403318

[25] van den Heuvel MP Sporns O. A cross-disorder connectome landscape of brain dysconnectivity. Nat Rev Neurosci. (2019) 20:435–46. doi: 10.1038/s41583-019-0177-6, 31127193 PMC8864539

[26] Yang Y Dai Y He Q Wang S Chen X Geng X . Altered brain functional connectivity in vegetative state and minimally conscious state. Front Aging Neurosci. (2023) 15:1213904. doi: 10.3389/fnagi.2023.1213904 PMC10352323 37469954

[27] Lynn CW Bassett DS. The physics of brain network structure, function and control. Nat Rev Phys. (2019) 1:318–32. doi: 10.1038/s42254-019-0040-8

[28] Plosnić G Raguž M Deletis V Chudy D. Dysfunctional connectivity as a neurophysiologic mechanism of disorders of consciousness: a systematic review. Front Neurosci. (2023) 17:1166187. doi: 10.3389/fnins.2023.1166187, 37539385 PMC10394244

[29] Cacciola A Naro A Milardi D Bramanti A Malatacca L Spitaleri M . Functional brain network topology discriminates between patients with minimally conscious state and unresponsive wakefulness syndrome. J Clin Med. (2019) 8:306. doi: 10.3390/jcm8030306, 30841486 PMC6463121

[30] Artoni F Delorme A Makeig S. Applying dimension reduction to EEG data by principal component analysis reduces the quality of its subsequent independent component decomposition. NeuroImage. (2018) 175:176–87. doi: 10.1016/j.neuroimage.2018.03.016, 29526744 PMC6650744

[31] Wu D-Y Cai G Yuan Y Liu L Li G-q Song W-q . Application of nonlinear dynamics analysis in assessing unconsciousness: a preliminary study. Clin Neurophysiol. (2011) 122:490–8. doi: 10.1016/j.clinph.2010.05.036, 20719560

[32] Engemann DA Raimondo F King J-R Rohaut B Louppe G Faugeras F . Robust EEG-based cross-site and cross-protocol classification of states of consciousness. Brain. (2018) 141:3179–92. doi: 10.1093/brain/awy251 30285102

[33] Piarulli A Bergamasco M Thibaut A Cologan V Gosseries O Laureys S. EEG ultradian rhythmicity differences in disorders of consciousness during wakefulness. J Neurol. (2016) 263:1746–60. doi: 10.1007/s00415-016-8196-y, 27294259

[34] Liu Y Zeng W Pan N Xia X Huang Y He J. EEG complexity correlates with residual consciousness level of disorders of consciousness. BMC Neurol. (2023) 23:140. doi: 10.1186/s12883-023-03167-w PMC10069047 37013466

[35] Cai L Wang J Guo Y Lu M Dong Y Wei X. Altered inter-frequency dynamics of brain networks in disorder of consciousness. J Neural Eng. (2020) 17:036006. doi: 10.1088/1741-2552/ab8b2c 32311694

[36] Chennu S Annen J Wannez S Thibaut A Chatelle C Cassol H . Brain networks predict metabolism, diagnosis and prognosis at the bedside in disorders of consciousness. Brain. (2017) 140:2120–32. doi: 10.1093/brain/awx163, 28666351

[37] Asghar MA Khan MJ Rizwan M Shorfuzzaman M Mehmood RM. AI inspired EEG-based spatial feature selection method using multivariate empirical mode decomposition for emotion classification. Multimed Syst. (2021) 28:1275–88. doi: 10.1007/s00530-021-00782-w, 33897112 PMC8057947

[38] Jain AK. Data clustering: 50 years beyond K-means. Pattern Recogn Lett. (2010) 31:651–66. doi: 10.1016/j.patrec.2009.09.011

[39] Hosseinifard B Moradi MH Rostami R. Classifying depression patients and normal subjects using machine learning techniques and nonlinear features from EEG signal. Comput Methods Prog Biomed. (2013) 109:339–45. doi: 10.1016/j.cmpb.2012.10.008 23122719

[40] Hosseini M.P. Nazem-Zadeh M.R. Mahmoudi F. Ying H. Soltanian-Zadeh H. (2014). Support vector machine with nonlinear-Kernel optimization for lateralization of epileptogenic hippocampus in MR Images, in: 2014 36th Annual International Conference of the IEEE Engineering in Medicine and Biology Society, Piscataway, NJ, USA: IEEE; 1047–1050. 10.1109/EMBC.2014.6943773 PMC4504209 25570141

[41] Memar P Faradji F. A novel multi-class EEG-based sleep stage classification system. IEEE Trans Neural Syst Rehabil Eng. (2018) 26:84–95. doi: 10.1109/TNSRE.2017.2776149 29324406

[42] Kingsford C Salzberg SL. What are decision trees? Nat Biotechnol. (2008) 26:1011–3. doi: 10.1038/nbt0908-1011, 18779814 PMC2701298

[43] Chiarelli AM Croce P Assenza G Merla A Granata G Giannantoni NM . Electroencephalography-derived prognosis of functional recovery in acute stroke through machine learning approaches. Int J Neural Syst. (2020) 30:2050067. doi: 10.1142/s0129065720500677 33236654

[44] Li H Dong L Su W Liu Y Tang Z Liao X . Multiple patterns of EEG parameters and their role in the prediction of patients with prolonged disorders of consciousness. Front Neurosci. (2025) 19:1492225. doi: 10.3389/fnins.2025.1492225, 39975972 PMC11836006

[45] Lawhern VJ Solon AJ Waytowich NR Gordon SM Hung CP Lance BJ. EEGNet: a compact convolutional neural network for EEG-based brain-computer interfaces. J Neural Eng. (2018) 15:056013. doi: 10.1088/1741-2552/aace8c, 29932424

[46] Annen J Mertel I Xu R Chatelle C Lesenfants D Ortner R . Auditory and somatosensory P3 are complementary for the assessment of patients with disorders of consciousness. Brain Sci. (2020) 10:748. doi: 10.3390/brainsci10100748, 33080842 PMC7602953

[47] Candia-Rivera D Annen J Gosseries O Thibaut A Laureys S Martial C. Neural responses to heartbeats detect residual signs of consciousness during resting state in postcomatose patients. J Neurosci. (2021) 41:5251–62. doi: 10.1523/JNEUROSCI.1740-20.2021, 33758019 PMC8211547

