# Supplementary material for: Auditory paradigm-dependent EEG feature screening and representation for distinguishing consciousness states in male patients with disorders of consciousness
Source: Front Neurol. 2026 Sep 30;17:1927313. doi: 10.3389/fneur.2026.1927313 (PMC13661486; doi:10.3389/fneur.2026.1927313)
Supplement: Supplementary file 1 [file Supplementary_file_1.docx]

Supplementary Material

Supplementary Table 1. Patient Information

| **No.** | **Age** | **Sex** | **Etiology** | **CRS-R** | **Diagnosis** | **Surgical Intervention** | **Time Since Injury** | **Medication** |
| --- | --- | --- | --- | --- | --- | --- | --- | --- |
| 1 | 50 | Male | ICH | 7 | UWS/VS | Heme evac, DC, Cranio | ~5 mo | GAL |
| 2 | 68 | Male | ICH | 11 | MCS | Heme evac, DC, Cranio | ~2–3 mo | LEV, VPA, Aman |
| 3 | 46 | Male | TBI | 17 | MCS | Craniot, Heme evac , Decomp | ~1 mo | GAL |
| 4 | 65 | Male | ICH | 2 | UWS/VS | DC, VP shunt, Cranio | ~4 mo | — |
| 5 | 53 | Male | ICH | 3 | UWS/VS | Heme evac, DC, Cranio | ~1–2 mo | CZP |
| 6 | 70 | Male | ICH | 7 | UWS/VS | Burr hole drain | ~11 mo (recur) | VPA, LEV, CZP |
| 7 | 53 | Male | ICH | 10 | MCS | Heme drain | ~2–3 mo | GAL / L-dopa |
| 8 | 51 | Male | TBI | 7 | UWS/VS | Heme evac, Decomp, VP shunt | ~7 mo | Aman |
| 9 | 69 | Male | ICH | 7 | MCS | Craniot, Heme evac, Decomp | ~2–3 mo | GAL |
| 10 | 44 | Male | ICH | 23 | MCS | Craniot, Heme evac, Decomp | ~1 mo | GAL |
| 11 | 69 | Male | TBI | 7 | UWS/VS | Craniot, Heme evac, Decomp | ~3 mo | GAL / L-dopa |
| 12 | 64 | Male | ICH | 7 | UWS/VS | Craniot, Decomp, Cranio, VP shunt | ~3 mo | Estazolam |
| 13 | 62 | Male | ICH | 4 | UWS/VS | Craniot, Heme evac, Decomp | ~1 mo | — |

Supplementary Continued Table 1. Patient Information

| **No.** | **Age** | **Sex** | **Etiology** | **CRS-R** | **Diagnosis** | **Surgical Intervention** | **Time Since Injury** | **Medication** |
| --- | --- | --- | --- | --- | --- | --- | --- | --- |
| 14 | 41 | Male | ICH | 2 | UWS/VS | EVD, Decomp, Heme evac, VP shunt | ~10 mo | — |
| 15 | 53 | Male | TBI | 9 | MCS | Craniot, Heme evac, Decomp | ~1 mo | — |

DC: Decompressive craniectomy ; Heme evac: Hematoma evacuation ; Cranio: Cranioplasty; Craniot: Craniotomy ; Decomp: Decompression ; VP shunt: Ventriculoperitoneal shunt ; EVD: External ventricular drainage ; Burr hole drain: Burr hole drainage ; mo:month ; recur: recurrent ; GAL: Galantamine ; LEV: Levetiracetam ; VPA: Valproate ; Aman: Amantadine ; CZP: Clonazepam ; L-dopa: Levodopa; ICH: Intracerebral hemorrhage; TBI: Traumatic brain injury

;

Supplementary Table 2. Statistical test results table for all features

| **Feature** | **SW_p** | **T_stat/U_stat** | **T_p/U_p** | **Cohens_d** |
| --- | --- | --- | --- | --- |
| LZC_D | 0.2107 | 2.4382 | 0.0448 | 1.4456 |
| LZC_N | 0.5096 | 2.4634 | 0.0389 | 1.4073 |
| LZC_R | 0.2649 | 3.1994 | 0.0145 | 1.8823 |
| LZC_T | 0.8060 | 2.3351 | 0.0404 | 1.2413 |
| SPE_D | 0.7424 | 2.5121 | 0.0298 | 1.3369 |
| SPE_N | 0.9441 | 2.5686 | 0.0261 | 1.3494 |
| SPE_R | 0.6147 | 3.3280 | 0.0073 | 1.7804 |
| SPE_T | 0.9860 | 2.6683 | 0.0217 | 1.3960 |
| PE_D | 0.3370 | 1.5900 | 0.1448 | 0.8720 |
| PE_N | 0.8506 | 1.6973 | 0.1136 | 0.8329 |
| PE_R | 0.6035 | 1.7427 | 0.1116 | 0.9369 |
| PE_T | 0.9659 | 1.1014 | 0.2926 | 0.5035 |
| SAE_D | 0.0823 | 2.3647 | 0.0521 | 1.4256 |
| SAE_N | 0.2156 | 2.3889 | 0.0470 | 1.4034 |
| SAE_R | 0.1230 | 3.1776 | 0.0162 | 1.9014 |
| SAE_T | 0.6066 | 2.3999 | 0.0382 | 1.3081 |
| AE_D | 0.0631 | 2.3458 | 0.0541 | 1.4207 |
| AE_N | 0.1726 | 2.3713 | 0.0491 | 1.4018 |
| AE_R | 0.0842 | 3.1187 | 0.0181 | 1.8780 |
| AE_T | 0.6140 | 2.4102 | 0.0375 | 1.3127 |
| PSD_Alpha_D | 0.0913 | 1.2644 | 0.2305 | 0.6492 |
| PSD_Alpha_N | 0.2501 | 1.4245 | 0.1790 | 0.7166 |
| PSD_Alpha_R | 0.1084 | 1.9404 | 0.0787 | 1.0223 |
| PSD_Alpha_T | 0.7520 | 2.2290 | 0.0441 | 1.0750 |
| PSD_Beta_D | 0.0063 | 40.0000 | 0.1447 | 1.1578 |
| PSD_Beta_N | 0.0037 | 43.0000 | 0.0663 | 1.1900 |
| PSD_Beta_R | 0.0030 | 49.0000 | 0.0076 | 1.4871 |
| PSD_Beta_T | 0.0477 | 45.0000 | 0.0360 | 1.2872 |
| PSD_Delta_D | 0.3069 | -2.2691 | 0.0423 | -1.1530 |
| PSD_Delta_N | 0.7299 | -2.4711 | 0.0284 | -1.2230 |
| PSD_Delta_R | 0.2472 | -2.8992 | 0.0150 | -1.5403 |
| PSD_Delta_T | 0.7065 | -2.7045 | 0.0185 | -1.3496 |
| PSD_Gamma_D | 0.0002 | 42.0000 | 0.0879 | 1.2622 |
| PSD_Gamma_N | 0.0012 | 46.0000 | 0.0256 | 1.0125 |
| PSD_Gamma_R | 0.0001 | 51.0000 | 0.0028 | 1.2829 |
| PSD_Gamma_T | 0.0092 | 40.0000 | 0.1447 | 0.5365 |
| PSD_Theta_D | 0.1763 | 1.1960 | 0.2637 | 0.6717 |
| PSD_Theta_N | 0.0897 | 1.1345 | 0.2881 | 0.6421 |
| PSD_Theta_R | 0.5284 | 1.4305 | 0.1936 | 0.8371 |
| PSD_Theta_T | 0.0975 | -1.4410 | 0.1938 | -0.8606 |
| WPLI_Alpha_Bet_D | 0.0613 | 1.5020 | 0.1584 | 0.6941 |
| WPLI_Alpha_Bet_N | 0.4030 | 0.7429 | 0.4728 | 0.3876 |
| WPLI_Alpha_Bet_R | 0.0979 | -0.2594 | 0.7994 | -0.1236 |
| WPLI_Alpha_Bet_T | 0.1365 | 0.8487 | 0.4202 | 0.4830 |
| WPLI_Alpha_De_D | 0.9428 | 1.0607 | 0.3083 | 0.5191 |
| WPLI_Alpha_De_N | 0.3397 | 1.7123 | 0.1107 | 0.8152 |
| WPLI_Alpha_De_R | 0.5123 | 0.8962 | 0.3867 | 0.4420 |
| WPLI_Alpha_De_T | 0.0736 | 2.1900 | 0.0523 | 1.1682 |
| WPLI_Alpha_Clo_D | 0.2680 | -0.9338 | 0.3690 | -0.4778 |
| WPLI_Alpha_Clo_N | 0.0147 | 18.0000 | 0.3277 | -0.6716 |
| WPLI_Alpha_Clo_R | 0.2353 | -1.2548 | 0.2317 | -0.6006 |
| WPLI_Alpha_Clo_T | 0.1207 | -2.6148 | 0.0215 | -1.2473 |
| WPLI_Alpha_CC_D | 0.9953 | 0.3767 | 0.7127 | 0.1751 |
| WPLI_Alpha_CC_N | 0.0812 | 0.6733 | 0.5198 | 0.3857 |
| WPLI_Alpha_CC_R | 0.5068 | -2.1453 | 0.0520 | -1.0027 |
| WPLI_Alpha_CC_T | 0.7598 | 2.1288 | 0.0613 | 1.1721 |
| WPLI_Beta_Bet_D | 0.7753 | 1.0726 | 0.3034 | 0.5332 |
| WPLI_Beta_Bet_N | 0.1799 | 1.5915 | 0.1380 | 0.8184 |
| WPLI_Beta_Bet_R | 0.2537 | 1.2915 | 0.2355 | 0.7554 |
| WPLI_Beta_Bet_T | 0.5015 | -0.1559 | 0.8790 | -0.0828 |
| WPLI_Beta_De_D | 0.0392 | 35.0000 | 0.3884 | 0.6010 |
| WPLI_Beta_De_N | 0.1204 | 1.8677 | 0.1042 | 1.1096 |
| WPLI_Beta_De_R | 0.2156 | 1.3160 | 0.2252 | 0.7566 |
| WPLI_Beta_De_T | 0.0034 | 42.0000 | 0.0879 | 1.0130 |
| WPLI_Beta_Clo_D | 0.8470 | -0.9197 | 0.3768 | -0.4779 |
| WPLI_Beta_Clo_N | 0.4449 | -1.6372 | 0.1367 | -0.9134 |
| WPLI_Beta_Clo_R | 0.0555 | -0.9131 | 0.3799 | -0.4729 |
| WPLI_Beta_Clo_T | 0.9969 | -2.0959 | 0.0643 | -1.1505 |
| WPLI_Beta_CC_D | 0.9709 | -1.1621 | 0.2661 | -0.5584 |
| WPLI_Beta_CC_N | 0.7701 | 0.3460 | 0.7352 | 0.1601 |
| WPLI_Beta_CC_R | 0.1420 | -1.5003 | 0.1575 | -0.7339 |
| WPLI_Beta_CC_T | 0.6115 | 1.3140 | 0.2133 | 0.6685 |
| WPLI_Delta_Bet_D | 0.1875 | 0.5347 | 0.6075 | 0.3070 |
| WPLI_Delta_Bet_N | 0.3354 | 1.6524 | 0.1316 | 0.9079 |
| WPLI_Delta_Bet_R | 0.1912 | -0.4978 | 0.6300 | -0.2728 |
| WPLI_Delta_Bet_T | 0.9123 | 1.2031 | 0.2553 | 0.6402 |
| WPLI_Delta_De_D | 0.7381 | 0.5326 | 0.6070 | 0.2945 |
| WPLI_Delta_De_N | 0.7089 | 1.7581 | 0.1139 | 0.9854 |
| WPLI_Delta_De_R | 0.2700 | -0.0944 | 0.9264 | -0.0434 |
| WPLI_Delta_De_T | 0.5756 | 1.5562 | 0.1566 | 0.8801 |
| WPLI_Delta_Clo_D | 0.0860 | -1.0574 | 0.3106 | -0.5330 |
| WPLI_Delta_Clo_N | 0.5697 | -1.2178 | 0.2473 | -0.6272 |
| WPLI_Delta_Clo_R | 0.6798 | -0.7989 | 0.4411 | -0.4179 |
| WPLI_Delta_Clo_T | 0.2339 | -1.7075 | 0.1133 | -0.8696 |
| WPLI_Delta_CC_D | 0.0493 | 33.0000 | 0.5287 | 0.7295 |
| WPLI_Delta_CC_N | 0.0103 | 36.0000 | 0.3277 | 0.8550 |
| WPLI_Delta_CC_R | 0.0970 | 1.4642 | 0.1881 | 0.8773 |
| WPLI_Delta_CC_T | 0.1811 | 0.6868 | 0.5074 | 0.3672 |
| WPLI_Gamma_Bet_D | 0.0233 | 21.0000 | 0.5161 | -0.1759 |
| WPLI_Gamma_Bet_N | 0.3364 | 0.2451 | 0.8116 | 0.1340 |
| WPLI_Gamma_Bet_R | 0.3549 | 0.5609 | 0.5848 | 0.2810 |
| WPLI_Gamma_Bet_T | 0.7149 | 0.4284 | 0.6798 | 0.2463 |
| WPLI_Gamma_De_D | 0.4613 | 0.4605 | 0.6593 | 0.2742 |
| WPLI_Gamma_De_N | 0.0032 | 26.0000 | 0.9546 | 0.2319 |
| WPLI_Gamma_De_R | 0.0622 | -0.3754 | 0.7138 | -0.1906 |
| WPLI_Gamma_De_T | 0.0020 | 22.0000 | 0.6070 | 0.2039 |
| WPLI_Gamma_Clo_D | 0.5482 | -0.2719 | 0.7920 | -0.1522 |
| WPLI_Gamma_Clo_N | 0.7277 | 0.3032 | 0.7703 | 0.1787 |
| WPLI_Gamma_Clo_R | 0.1356 | 0.8562 | 0.4120 | 0.4623 |
| WPLI_Gamma_Clo_T | 0.8424 | 0.4395 | 0.6767 | 0.2781 |
| WPLI_Gamma_CC_D | 0.3374 | 0.1622 | 0.8737 | 0.0764 |
| WPLI_Gamma_CC_N | 0.4956 | -0.5696 | 0.5855 | -0.3309 |
| WPLI_Gamma_CC_R | 0.1276 | -0.3410 | 0.7417 | -0.1940 |
| WPLI_Gamma_CC_T | 0.3713 | 0.0741 | 0.9422 | 0.0384 |
| WPLI_Theta_Bet_D | 0.5745 | 1.1383 | 0.2848 | 0.6338 |
| WPLI_Theta_Bet_N | 0.1967 | 0.5809 | 0.5756 | 0.3226 |
| WPLI_Theta_Bet_R | 0.5130 | 1.2835 | 0.2384 | 0.7518 |
| WPLI_Theta_Bet_T | 0.7827 | -0.1202 | 0.9068 | -0.0660 |
| WPLI_Theta_De_D | 0.2158 | 0.4326 | 0.6789 | 0.2602 |
| WPLI_Theta_De_N | 0.9604 | 1.3474 | 0.2118 | 0.7541 |
| WPLI_Theta_De_R | 0.6899 | 1.0012 | 0.3438 | 0.5608 |
| WPLI_Theta_De_T | 0.4164 | 2.3344 | 0.0365 | 1.1056 |
| WPLI_Theta_Clo_D | 0.1195 | -0.1002 | 0.9221 | -0.0537 |
| WPLI_Theta_Clo_N | 0.4048 | -1.7692 | 0.1005 | -0.8395 |
| WPLI_Theta_Clo_R | 0.2343 | -0.7632 | 0.4600 | -0.3504 |
| WPLI_Theta_Clo_T | 0.0161 | 9.0000 | 0.0360 | -1.6391 |
| WPLI_Theta_CC_D | 0.2655 | 0.4225 | 0.6858 | 0.2534 |
| WPLI_Theta_CC_N | 0.0563 | 0.1370 | 0.8937 | 0.0737 |
| WPLI_Theta_CC_R | 0.0981 | 0.0534 | 0.9586 | 0.0293 |
| WPLI_Theta_CC_T | 0.4889 | 1.0859 | 0.3034 | 0.5885 |
| WPLI_Band_Bet_D | 0.4000 | 0.6591 | 0.5327 | 0.4006 |
| WPLI_Band_Bet_N | 0.1386 | 0.9781 | 0.3644 | 0.5994 |
| WPLI_Band_Bet_R | 0.8733 | 0.5654 | 0.5872 | 0.3225 |
| WPLI_Band_Bet_T | 0.9950 | 0.1481 | 0.8868 | 0.0905 |
| WPLI_Band_De_D | 0.1945 | 1.3187 | 0.2180 | 0.7206 |
| WPLI_Band_De_N | 0.8645 | 2.0408 | 0.0771 | 1.1808 |
| WPLI_Band_De_R | 0.6074 | 1.0782 | 0.3044 | 0.5691 |
| WPLI_Band_De_T | 0.2250 | 2.0096 | 0.0880 | 1.2214 |
| WPLI_Band_Clo_D | 0.0162 | 17.0000 | 0.2721 | -0.5681 |
| WPLI_Band_Clo_N | 0.1544 | -1.8766 | 0.0847 | -0.9520 |
| WPLI_Band_Clo_R | 0.0958 | -1.0117 | 0.3313 | -0.5123 |
| WPLI_Band_Clo_T | 0.4713 | -1.8760 | 0.0905 | -1.0154 |
| WPLI_Band_CC_D | 0.7078 | -0.4903 | 0.6323 | -0.2298 |
| WPLI_Band_CC_N | 0.8914 | -0.8706 | 0.3999 | -0.4281 |
| WPLI_Band_CC_r | 0.7839 | -1.0246 | 0.3388 | -0.6035 |
| WPLI_Band_CC_T | 0.7302 | 1.7255 | 0.1083 | 0.8195 |

WPLI: Weighted Phase Lag Index; De: Degree; CC: Clustering Coefficient; Clo: Closeness Centrality; Bet: Betweenness Centrality; PSD: Power Spectral Density; PE: Permutation Entropy; AE: Approximate Entropy; SAE: Sample Entropy; SPE: Spectral Entropy; LZC: Lempel-Ziv Complexity; Band: Full Frequency Band; T: Pure-tone Paradigm; N: Name-calling Paradigm; R: Reversed-word Sequence Paradigm; D: Different-word Sequence Paradigm

Supplementary Table 3. Table of classifier results for all single features

| **Feature** | **Model** | **Accuracy** | **Precision** | **Recall** | **Kappa** | **AUC** | |
| --- | --- | --- | --- | --- | --- | --- | --- |
| LZC_D | DT | 0.8667 | 0.7500 | 1.0000 | 0.7368 | 0.8889 |  |
|  | KNN | 0.6667 | 0.6667 | 0.3333 | 0.2424 | 0.4630 |  |
|  | LR | 0.6000 | 0.0000 | 0.0000 | 0.0000 | 0.0000 |  |
|  | RF | 0.8667 | 0.7500 | 1.0000 | 0.7368 | 0.8426 |  |
|  | SVM | 0.6000 | 0.0000 | 0.0000 | 0.0000 | 0.0000 |  |
| LZC_N | DT | 0.5333 | 0.4000 | 0.3333 | 0.0000 | 0.5000 |  |
|  | KNN | 0.7333 | 0.6667 | 0.6667 | 0.4444 | 0.6296 |  |
|  | LR | 0.6000 | 0.0000 | 0.0000 | 0.0000 | 0.0000 |  |
|  | RF | 0.5333 | 0.4000 | 0.3333 | 0.0000 | 0.5000 |  |
|  | SVM | 0.6000 | 0.0000 | 0.0000 | 0.0000 | 0.0000 |  |
| LZC_R | DT | 0.6667 | 0.5714 | 0.6667 | 0.3243 | 0.6667 |  |
|  | KNN | 0.8667 | 0.8333 | 0.8333 | 0.7222 | 0.7500 |  |
|  | LR | 0.6000 | 0.0000 | 0.0000 | 0.0000 | 0.0000 |  |
|  | RF | 0.6667 | 0.5714 | 0.6667 | 0.3243 | 0.7222 |  |
|  | SVM | 0.6000 | 0.0000 | 0.0000 | 0.0000 | 0.0000 |  |
| LZC_T | DT | 0.5333 | 0.4286 | 0.5000 | 0.0541 | 0.5278 |  |
|  | KNN | 0.7333 | 0.6667 | 0.6667 | 0.4444 | 0.6111 |  |
|  | LR | 0.6000 | 0.0000 | 0.0000 | 0.0000 | 0.0000 |  |
|  | RF | 0.5333 | 0.4286 | 0.5000 | 0.0541 | 0.6296 |  |
|  | SVM | 0.6000 | 0.0000 | 0.0000 | 0.0000 | 0.0000 |  |
| SPE_D | DT | 0.6667 | 0.5714 | 0.6667 | 0.3243 | 0.6667 |  |
|  | KNN | 0.6667 | 0.6667 | 0.3333 | 0.2424 | 0.5556 |  |
|  | LR | 0.6000 | 0.5000 | 0.1667 | 0.0625 | 0.6481 |  |
|  | RF | 0.6667 | 0.5714 | 0.6667 | 0.3243 | 0.6667 |  |
|  | SVM | 0.5333 | 0.0000 | 0.0000 | -0.1290 | 0.3704 |  |
| SPE_N | DT | 0.5333 | 0.4000 | 0.3333 | 0.0000 | 0.5000 |  |
|  | KNN | 0.8000 | 0.8000 | 0.6667 | 0.5714 | 0.7222 |  |
|  | LR | 0.6000 | 0.5000 | 0.1667 | 0.0625 | 0.6481 |  |
|  | RF | 0.5333 | 0.4000 | 0.3333 | 0.0000 | 0.6111 |  |
|  | SVM | 0.5333 | 0.0000 | 0.0000 | -0.1290 | 0.2593 |  |
| SPE_R | DT | 0.7333 | 0.6667 | 0.6667 | 0.4444 | 0.7222 |  |
|  | KNN | 0.8000 | 0.8000 | 0.6667 | 0.5714 | 0.7685 |  |
|  | LR | 0.6667 | 0.6667 | 0.3333 | 0.2424 | 0.7778 |  |
|  | RF | 0.7333 | 0.6667 | 0.6667 | 0.4444 | 0.6389 |  |
|  | SVM | 0.8000 | 0.8000 | 0.6667 | 0.5714 | 0.7222 |  |
| SPE_T | DT | 0.6667 | 0.6000 | 0.5000 | 0.2857 | 0.6389 |  |
|  | KNN | 0.7333 | 0.7500 | 0.5000 | 0.4118 | 0.6019 |  |
|  | LR | 0.6000 | 0.0000 | 0.0000 | 0.0000 | 0.5185 |  |
|  | RF | 0.6667 | 0.6000 | 0.5000 | 0.2857 | 0.6019 |  |
|  | SVM | 0.6000 | 0.0000 | 0.0000 | 0.0000 | 0.0926 |  |
| PE_D | DT | 0.6667 | 0.6000 | 0.5000 | 0.2857 | 0.6389 |  |
|  | KNN | 0.2000 | 0.0000 | 0.0000 | -0.6667 | 0.3056 |  |
|  | LR | 0.6000 | 0.0000 | 0.0000 | 0.0000 | 0.0000 |  |
|  | RF | 0.6667 | 0.6000 | 0.5000 | 0.2857 | 0.4815 |  |
|  | SVM | 0.6000 | 0.0000 | 0.0000 | 0.0000 | 0.0000 |  |
| PE_N | DT | 0.6000 | 0.5000 | 0.3333 | 0.1176 | 0.5556 |  |
|  | KNN | 0.4000 | 0.2000 | 0.1667 | -0.2857 | 0.4815 |  |
|  | LR | 0.6000 | 0.0000 | 0.0000 | 0.0000 | 0.0000 |  |
|  | RF | 0.6000 | 0.5000 | 0.3333 | 0.1176 | 0.5648 |  |
|  | SVM | 0.6000 | 0.0000 | 0.0000 | 0.0000 | 0.0000 |  |
| PE_R | DT | 0.8000 | 0.7143 | 0.8333 | 0.5946 | 0.8056 |  |
|  | KNN | 0.4000 | 0.2857 | 0.3333 | -0.2162 | 0.2593 |  |
|  | LR | 0.6000 | 0.0000 | 0.0000 | 0.0000 | 0.0000 |  |
|  | RF | 0.8000 | 0.7143 | 0.8333 | 0.5946 | 0.7407 |  |
|  | SVM | 0.6000 | 0.0000 | 0.0000 | 0.0000 | 0.0000 |  |
| PE_T | DT | 0.4667 | 0.3750 | 0.5000 | -0.0526 | 0.4722 |  |
|  | KNN | 0.5333 | 0.4444 | 0.6667 | 0.1026 | 0.5185 |  |
|  | LR | 0.6000 | 0.0000 | 0.0000 | 0.0000 | 0.0000 |  |
|  | RF | 0.4667 | 0.3750 | 0.5000 | -0.0526 | 0.6667 |  |
|  | SVM | 0.6000 | 0.0000 | 0.0000 | 0.0000 | 0.0000 |  |
| SAE_D | DT | 0.8667 | 0.7500 | 1.0000 | 0.7368 | 0.8889 |  |
|  | KNN | 0.6667 | 0.6667 | 0.3333 | 0.2424 | 0.4630 |  |
|  | LR | 0.6000 | 0.0000 | 0.0000 | 0.0000 | 0.0000 |  |
|  | RF | 0.8667 | 0.7500 | 1.0000 | 0.7368 | 0.8333 |  |
|  | SVM | 0.6000 | 0.0000 | 0.0000 | 0.0000 | 0.0000 |  |
| SAE_N | DT | 0.5333 | 0.4000 | 0.3333 | 0.0000 | 0.5000 |  |
|  | KNN | 0.7333 | 0.6667 | 0.6667 | 0.4444 | 0.6296 |  |
|  | LR | 0.6000 | 0.0000 | 0.0000 | 0.0000 | 0.0000 |  |
|  | RF | 0.5333 | 0.4000 | 0.3333 | 0.0000 | 0.4815 |  |
|  | SVM | 0.6000 | 0.0000 | 0.0000 | 0.0000 | 0.0000 |  |
| SAE_R | DT | 0.7333 | 0.6667 | 0.6667 | 0.4444 | 0.7222 |  |
|  | KNN | 0.8667 | 0.8333 | 0.8333 | 0.7222 | 0.7500 |  |
|  | LR | 0.6000 | 0.0000 | 0.0000 | 0.0000 | 0.0000 |  |
|  | RF | 0.7333 | 0.6667 | 0.6667 | 0.4444 | 0.7407 |  |
|  | SVM | 0.6000 | 0.0000 | 0.0000 | 0.0000 | 0.0000 |  |
| SAE_T | DT | 0.5333 | 0.4000 | 0.3333 | 0.0000 | 0.5000 |  |
|  | KNN | 0.7333 | 0.6667 | 0.6667 | 0.4444 | 0.6111 |  |
|  | LR | 0.6000 | 0.0000 | 0.0000 | 0.0000 | 0.0000 |  |
|  | RF | 0.5333 | 0.4000 | 0.3333 | 0.0000 | 0.6019 |  |
|  | SVM | 0.6000 | 0.0000 | 0.0000 | 0.0000 | 0.0000 |  |
| AE_D | DT | 0.6667 | 0.5714 | 0.6667 | 0.3243 | 0.6667 |  |
|  | KNN | 0.6667 | 0.6667 | 0.3333 | 0.2424 | 0.4630 |  |
|  | LR | 0.6000 | 0.0000 | 0.0000 | 0.0000 | 0.0000 |  |
|  | RF | 0.6667 | 0.5714 | 0.6667 | 0.3243 | 0.7222 |  |
|  | SVM | 0.6000 | 0.0000 | 0.0000 | 0.0000 | 0.0000 |  |
| AE_N | DT | 0.5333 | 0.4000 | 0.3333 | 0.0000 | 0.5000 |  |
|  | KNN | 0.6667 | 0.6000 | 0.5000 | 0.2857 | 0.5926 |  |
|  | LR | 0.6000 | 0.0000 | 0.0000 | 0.0000 | 0.0000 |  |
|  | RF | 0.5333 | 0.4000 | 0.3333 | 0.0000 | 0.4815 |  |
|  | SVM | 0.6000 | 0.0000 | 0.0000 | 0.0000 | 0.0000 |  |
| AE_R | DT | 0.7333 | 0.6667 | 0.6667 | 0.4444 | 0.7222 |  |
|  | KNN | 0.8667 | 0.8333 | 0.8333 | 0.7222 | 0.7500 |  |
|  | LR | 0.6000 | 0.0000 | 0.0000 | 0.0000 | 0.0000 |  |
|  | RF | 0.7333 | 0.6667 | 0.6667 | 0.4444 | 0.7407 |  |
|  | SVM | 0.6000 | 0.0000 | 0.0000 | 0.0000 | 0.0556 |  |
| AE_T | DT | 0.6000 | 0.5000 | 0.3333 | 0.1176 | 0.5556 |  |
|  | KNN | 0.7333 | 0.6667 | 0.6667 | 0.4444 | 0.6111 |  |
|  | LR | 0.6000 | 0.0000 | 0.0000 | 0.0000 | 0.0000 |  |
|  | RF | 0.6000 | 0.5000 | 0.3333 | 0.1176 | 0.6667 |  |
|  | SVM | 0.6000 | 0.0000 | 0.0000 | 0.0000 | 0.0000 |  |
| PSD_Alpha_D | DT | 0.4667 | 0.3333 | 0.3333 | -0.1111 | 0.4444 |  |
|  | KNN | 0.5333 | 0.4000 | 0.3333 | 0.0000 | 0.4444 |  |
|  | LR | 0.6000 | 0.0000 | 0.0000 | 0.0000 | 0.0000 |  |
|  | RF | 0.4667 | 0.3333 | 0.3333 | -0.1111 | 0.3241 |  |
|  | SVM | 0.6000 | 0.0000 | 0.0000 | 0.0000 | 0.0000 |  |
| PSD_Alpha_N | DT | 0.6000 | 0.5000 | 0.5000 | 0.1667 | 0.5833 |  |
|  | KNN | 0.4667 | 0.3333 | 0.3333 | -0.1111 | 0.4074 |  |
|  | LR | 0.6000 | 0.0000 | 0.0000 | 0.0000 | 0.0000 |  |
|  | RF | 0.6000 | 0.5000 | 0.5000 | 0.1667 | 0.5556 |  |
|  | SVM | 0.6000 | 0.0000 | 0.0000 | 0.0000 | 0.0000 |  |
| PSD_Alpha_R | DT | 0.6667 | 0.6000 | 0.5000 | 0.2857 | 0.6389 |  |
|  | KNN | 0.6667 | 0.6000 | 0.5000 | 0.2857 | 0.5370 |  |
|  | LR | 0.6000 | 0.0000 | 0.0000 | 0.0000 | 0.0000 |  |
|  | RF | 0.6667 | 0.6000 | 0.5000 | 0.2857 | 0.7222 |  |
|  | SVM | 0.6000 | 0.0000 | 0.0000 | 0.0000 | 0.0000 |  |
| PSD_Alpha_T | DT | 0.5333 | 0.4000 | 0.3333 | 0.0000 | 0.5000 |  |
|  | KNN | 0.6667 | 0.5714 | 0.6667 | 0.3243 | 0.6481 |  |
|  | LR | 0.6000 | 0.0000 | 0.0000 | 0.0000 | 0.0000 |  |
|  | RF | 0.5333 | 0.4000 | 0.3333 | 0.0000 | 0.5556 |  |
|  | SVM | 0.6000 | 0.0000 | 0.0000 | 0.0000 | 0.0000 |  |
| PSD_Beta_D | DT | 0.5333 | 0.4286 | 0.5000 | 0.0541 | 0.5278 |  |
|  | KNN | 0.6667 | 0.6667 | 0.3333 | 0.2424 | 0.5741 |  |
|  | LR | 0.6000 | 0.0000 | 0.0000 | 0.0000 | 0.0000 |  |
|  | RF | 0.5333 | 0.4286 | 0.5000 | 0.0541 | 0.4259 |  |
|  | SVM | 0.6000 | 0.0000 | 0.0000 | 0.0000 | 0.0000 |  |
| PSD_Beta_N | DT | 0.6000 | 0.5000 | 0.3333 | 0.1176 | 0.5556 |  |
|  | KNN | 0.6667 | 1.0000 | 0.1667 | 0.1935 | 0.3426 |  |
|  | LR | 0.6000 | 0.0000 | 0.0000 | 0.0000 | 0.0000 |  |
|  | RF | 0.6000 | 0.5000 | 0.3333 | 0.1176 | 0.3981 |  |
|  | SVM | 0.6000 | 0.0000 | 0.0000 | 0.0000 | 0.0000 |  |
| PSD_Beta_R | DT | 0.6000 | 0.5000 | 0.5000 | 0.1667 | 0.5833 |  |
|  | KNN | 0.8000 | 0.8000 | 0.6667 | 0.5714 | 0.7407 |  |
|  | LR | 0.6000 | 0.0000 | 0.0000 | 0.0000 | 0.0000 |  |
|  | RF | 0.6000 | 0.5000 | 0.5000 | 0.1667 | 0.6667 |  |
|  | SVM | 0.6000 | 0.0000 | 0.0000 | 0.0000 | 0.4815 |  |
| PSD_Beta_T | DT | 0.5333 | 0.4286 | 0.5000 | 0.0541 | 0.5278 |  |
|  | KNN | 0.6667 | 0.6000 | 0.5000 | 0.2857 | 0.4815 |  |
|  | LR | 0.6000 | 0.0000 | 0.0000 | 0.0000 | 0.0000 |  |
|  | RF | 0.5333 | 0.4286 | 0.5000 | 0.0541 | 0.4630 |  |
|  | SVM | 0.6000 | 0.0000 | 0.0000 | 0.0000 | 0.0000 |  |
| PSD_Delta_D | DT | 0.6667 | 0.5714 | 0.6667 | 0.3243 | 0.6667 |  |
|  | KNN | 0.6667 | 0.6000 | 0.5000 | 0.2857 | 0.5370 |  |
|  | LR | 0.6000 | 0.0000 | 0.0000 | 0.0000 | 0.0000 |  |
|  | RF | 0.6667 | 0.5714 | 0.6667 | 0.3243 | 0.6019 |  |
|  | SVM | 0.6000 | 0.0000 | 0.0000 | 0.0000 | 0.0000 |  |
| PSD_Delta_N | DT | 0.6000 | 0.5000 | 0.3333 | 0.1176 | 0.5556 |  |
|  | KNN | 0.7333 | 0.6667 | 0.6667 | 0.4444 | 0.6667 |  |
|  | LR | 0.6000 | 0.0000 | 0.0000 | 0.0000 | 0.0000 |  |
|  | RF | 0.6000 | 0.5000 | 0.3333 | 0.1176 | 0.6019 |  |
|  | SVM | 0.6000 | 0.0000 | 0.0000 | 0.0000 | 0.0000 |  |
| PSD_Delta_R | DT | 0.6000 | 0.5000 | 0.5000 | 0.1667 | 0.5833 |  |
|  | KNN | 0.8000 | 0.8000 | 0.6667 | 0.5714 | 0.6389 |  |
|  | LR | 0.6000 | 0.0000 | 0.0000 | 0.0000 | 0.0000 |  |
|  | RF | 0.6000 | 0.5000 | 0.5000 | 0.1667 | 0.5833 |  |
|  | SVM | 0.6000 | 0.0000 | 0.0000 | 0.0000 | 0.0000 |  |
| PSD_Delta_T | DT | 0.5333 | 0.4444 | 0.6667 | 0.1026 | 0.5556 |  |
|  | KNN | 0.6000 | 0.5000 | 0.5000 | 0.1667 | 0.6389 |  |
|  | LR | 0.6000 | 0.0000 | 0.0000 | 0.0000 | 0.0000 |  |
|  | RF | 0.5333 | 0.4444 | 0.6667 | 0.1026 | 0.5370 |  |
|  | SVM | 0.6000 | 0.0000 | 0.0000 | 0.0000 | 0.0000 |  |
| PSD_Gamma_D | DT | 0.4667 | 0.3333 | 0.3333 | -0.1111 | 0.4444 |  |
|  | KNN | 0.4667 | 0.0000 | 0.0000 | -0.2500 | 0.3056 |  |
|  | LR | 0.6000 | 0.0000 | 0.0000 | 0.0000 | 0.0000 |  |
|  | RF | 0.4667 | 0.3333 | 0.3333 | -0.1111 | 0.3519 |  |
|  | SVM | 0.6000 | 0.0000 | 0.0000 | 0.0000 | 0.5000 |  |
| PSD_Gamma_N | DT | 0.7333 | 0.6667 | 0.6667 | 0.4444 | 0.7222 |  |
|  | KNN | 0.7333 | 0.6667 | 0.6667 | 0.4444 | 0.7593 |  |
|  | LR | 0.6000 | 0.0000 | 0.0000 | 0.0000 | 0.0000 |  |
|  | RF | 0.7333 | 0.6667 | 0.6667 | 0.4444 | 0.8148 |  |
|  | SVM | 0.6000 | 0.0000 | 0.0000 | 0.0000 | 0.0000 |  |
| PSD_Gamma_R | DT | 0.8000 | 0.8000 | 0.6667 | 0.5714 | 0.7778 |  |
|  | KNN | 0.7333 | 0.7500 | 0.5000 | 0.4118 | 0.7593 |  |
|  | LR | 0.6000 | 0.0000 | 0.0000 | 0.0000 | 0.0000 |  |
|  | RF | 0.8000 | 0.8000 | 0.6667 | 0.5714 | 0.8241 |  |
|  | SVM | 0.6000 | 0.0000 | 0.0000 | 0.0000 | 0.5000 |  |
| PSD_Gamma_T | DT | 0.4667 | 0.3333 | 0.3333 | -0.1111 | 0.4444 |  |
|  | KNN | 0.6000 | 0.5000 | 0.6667 | 0.2105 | 0.5926 |  |
|  | LR | 0.6000 | 0.0000 | 0.0000 | 0.0000 | 0.0000 |  |
|  | RF | 0.4667 | 0.3333 | 0.3333 | -0.1111 | 0.5741 |  |
|  | SVM | 0.6000 | 0.0000 | 0.0000 | 0.0000 | 0.1667 |  |
| PSD_Theta_D | DT | 0.5333 | 0.4444 | 0.6667 | 0.1026 | 0.5556 |  |
|  | KNN | 0.4667 | 0.0000 | 0.0000 | -0.2500 | 0.4630 |  |
|  | LR | 0.6000 | 0.0000 | 0.0000 | 0.0000 | 0.0000 |  |
|  | RF | 0.5333 | 0.4444 | 0.6667 | 0.1026 | 0.3148 |  |
|  | SVM | 0.6000 | 0.0000 | 0.0000 | 0.0000 | 0.1667 |  |
| PSD_Theta_N | DT | 0.6667 | 0.5714 | 0.6667 | 0.3243 | 0.6667 |  |
|  | KNN | 0.6000 | 0.0000 | 0.0000 | 0.0000 | 0.3148 |  |
|  | LR | 0.6000 | 0.0000 | 0.0000 | 0.0000 | 0.0000 |  |
|  | RF | 0.6667 | 0.5714 | 0.6667 | 0.3243 | 0.5926 |  |
|  | SVM | 0.6000 | 0.0000 | 0.0000 | 0.0000 | 0.1667 |  |
| PSD_Theta_R | DT | 0.6000 | 0.5000 | 0.5000 | 0.1667 | 0.5833 |  |
|  | KNN | 0.6000 | 0.0000 | 0.0000 | 0.0000 | 0.3056 |  |
|  | LR | 0.6000 | 0.0000 | 0.0000 | 0.0000 | 0.0000 |  |
|  | RF | 0.6000 | 0.5000 | 0.5000 | 0.1667 | 0.5370 |  |
|  | SVM | 0.6000 | 0.0000 | 0.0000 | 0.0000 | 0.1667 |  |
| PSD_Theta_T | DT | 0.6000 | 0.5000 | 0.5000 | 0.1667 | 0.5833 |  |
|  | KNN | 0.6000 | 0.5000 | 0.3333 | 0.1176 | 0.4537 |  |
|  | LR | 0.6000 | 0.0000 | 0.0000 | 0.0000 | 0.0000 |  |
|  | RF | 0.6000 | 0.5000 | 0.5000 | 0.1667 | 0.4630 |  |
|  | SVM | 0.6000 | 0.0000 | 0.0000 | 0.0000 | 0.0000 |  |
| WPLI_Alpha_Bet_D | DT | 0.4000 | 0.2857 | 0.3333 | -0.2162 | 0.3889 |  |
|  | KNN | 0.4667 | 0.3750 | 0.5000 | -0.0526 | 0.4444 |  |
|  | LR | 0.6000 | 0.0000 | 0.0000 | 0.0000 | 0.0000 |  |
|  | RF | 0.4000 | 0.2857 | 0.3333 | -0.2162 | 0.4074 |  |
|  | SVM | 0.6000 | 0.0000 | 0.0000 | 0.0000 | 0.0000 |  |
| WPLI_Alpha_Bet_N | DT | 0.3333 | 0.1667 | 0.1667 | -0.3889 | 0.3056 |  |
|  | KNN | 0.2667 | 0.0000 | 0.0000 | -0.5714 | 0.1852 |  |
|  | LR | 0.6000 | 0.0000 | 0.0000 | 0.0000 | 0.0000 |  |
|  | RF | 0.3333 | 0.1667 | 0.1667 | -0.3889 | 0.0741 |  |
|  | SVM | 0.6000 | 0.0000 | 0.0000 | 0.0000 | 0.0000 |  |
| WPLI_Alpha_Bet_R | DT | 0.6667 | 0.6000 | 0.5000 | 0.2857 | 0.7037 |  |
|  | KNN | 0.6000 | 0.5000 | 0.3333 | 0.1176 | 0.3889 |  |
|  | LR | 0.6000 | 0.0000 | 0.0000 | 0.0000 | 0.0000 |  |
|  | RF | 0.7333 | 0.6667 | 0.6667 | 0.4444 | 0.6111 |  |
|  | SVM | 0.6000 | 0.0000 | 0.0000 | 0.0000 | 0.1667 |  |
| WPLI_Alpha_Bet_T | DT | 0.6667 | 0.6000 | 0.5000 | 0.2857 | 0.6389 |  |
|  | KNN | 0.6000 | 0.5000 | 0.3333 | 0.1176 | 0.3981 |  |
|  | LR | 0.6000 | 0.0000 | 0.0000 | 0.0000 | 0.0000 |  |
|  | RF | 0.6667 | 0.6000 | 0.5000 | 0.2857 | 0.5926 |  |
|  | SVM | 0.6000 | 0.0000 | 0.0000 | 0.0000 | 0.0000 |  |
| WPLI_Alpha_De_D | DT | 0.5333 | 0.4286 | 0.5000 | 0.0541 | 0.5278 |  |
|  | KNN | 0.4667 | 0.3333 | 0.3333 | -0.1111 | 0.3426 |  |
|  | LR | 0.4667 | 0.0000 | 0.0000 | -0.2500 | 0.4444 |  |
|  | RF | 0.5333 | 0.4286 | 0.5000 | 0.0541 | 0.4630 |  |
|  | SVM | 0.4667 | 0.0000 | 0.0000 | -0.2500 | 0.4444 |  |
| WPLI_Alpha_De_N | DT | 0.6000 | 0.5000 | 0.5000 | 0.1667 | 0.5833 |  |
|  | KNN | 0.6667 | 0.5714 | 0.6667 | 0.3243 | 0.6296 |  |
|  | LR | 0.5333 | 0.3333 | 0.1667 | -0.0606 | 0.6111 |  |
|  | RF | 0.6000 | 0.5000 | 0.5000 | 0.1667 | 0.6019 |  |
|  | SVM | 0.3333 | 0.0000 | 0.0000 | -0.4706 | 0.4630 |  |
| WPLI_Alpha_De_R | DT | 0.6000 | 0.5000 | 0.6667 | 0.2105 | 0.6111 |  |
|  | KNN | 0.3333 | 0.1667 | 0.1667 | -0.3889 | 0.4167 |  |
|  | LR | 0.5333 | 0.0000 | 0.0000 | -0.1290 | 0.4074 |  |
|  | RF | 0.6000 | 0.5000 | 0.6667 | 0.2105 | 0.5185 |  |
|  | SVM | 0.3333 | 0.0000 | 0.0000 | -0.4706 | 0.4815 |  |
| WPLI_Alpha_De_T | DT | 0.6000 | 0.5000 | 0.5000 | 0.1667 | 0.5833 |  |
|  | KNN | 0.8000 | 1.0000 | 0.5000 | 0.5455 | 0.6759 |  |
|  | LR | 0.6667 | 0.5714 | 0.6667 | 0.3243 | 0.6667 |  |
|  | RF | 0.6000 | 0.5000 | 0.5000 | 0.1667 | 0.5741 |  |
|  | SVM | 0.6000 | 0.5000 | 0.6667 | 0.2105 | 0.1852 |  |
| WPLI_Alpha_Clo_D | DT | 0.4000 | 0.3333 | 0.5000 | -0.1538 | 0.4167 |  |
|  | KNN | 0.3333 | 0.0000 | 0.0000 | -0.4706 | 0.2778 |  |
|  | LR | 0.4667 | 0.0000 | 0.0000 | -0.2500 | 0.4259 |  |
|  | RF | 0.4000 | 0.3333 | 0.5000 | -0.1538 | 0.2407 |  |
|  | SVM | 0.4000 | 0.0000 | 0.0000 | -0.3636 | 0.5463 |  |
| WPLI_Alpha_Clo_N | DT | 0.5333 | 0.4000 | 0.3333 | 0.0000 | 0.5000 |  |
|  | KNN | 0.2000 | 0.0000 | 0.0000 | -0.6667 | 0.3056 |  |
|  | LR | 0.4667 | 0.2500 | 0.1667 | -0.1765 | 0.4630 |  |
|  | RF | 0.5333 | 0.4000 | 0.3333 | 0.0000 | 0.3148 |  |
|  | SVM | 0.2000 | 0.0000 | 0.0000 | -0.6667 | 0.8148 |  |
| WPLI_Alpha_Clo_R | DT | 0.2667 | 0.1429 | 0.1667 | -0.4865 | 0.2500 |  |
|  | KNN | 0.5333 | 0.4444 | 0.6667 | 0.1026 | 0.4630 |  |
|  | LR | 0.4000 | 0.0000 | 0.0000 | -0.3636 | 0.4444 |  |
|  | RF | 0.2667 | 0.1429 | 0.1667 | -0.4865 | 0.2407 |  |
|  | SVM | 0.2667 | 0.0000 | 0.0000 | -0.5714 | 0.2778 |  |
| WPLI_Alpha_Clo_T | DT | 0.6000 | 0.5000 | 0.3333 | 0.1176 | 0.5556 |  |
|  | KNN | 0.6000 | 0.5000 | 0.5000 | 0.1667 | 0.5833 |  |
|  | LR | 0.6667 | 0.5714 | 0.6667 | 0.3243 | 0.7037 |  |
|  | RF | 0.6000 | 0.5000 | 0.3333 | 0.1176 | 0.5185 |  |
|  | SVM | 0.6667 | 0.5714 | 0.6667 | 0.3243 | 0.3704 |  |
| WPLI_Alpha_CC_D | DT | 0.4000 | 0.2857 | 0.3333 | -0.2162 | 0.3889 |  |
|  | KNN | 0.5333 | 0.4286 | 0.5000 | 0.0541 | 0.4537 |  |
|  | LR | 0.6000 | 0.0000 | 0.0000 | 0.0000 | 0.0000 |  |
|  | RF | 0.4000 | 0.2857 | 0.3333 | -0.2162 | 0.3704 |  |
|  | SVM | 0.6000 | 0.0000 | 0.0000 | 0.0000 | 0.0000 |  |
| WPLI_Alpha_CC_N | DT | 0.3333 | 0.0000 | 0.0000 | -0.4706 | 0.2778 |  |
|  | KNN | 0.4000 | 0.0000 | 0.0000 | -0.3636 | 0.1111 |  |
|  | LR | 0.6000 | 0.0000 | 0.0000 | 0.0000 | 0.0000 |  |
|  | RF | 0.3333 | 0.0000 | 0.0000 | -0.4706 | 0.0000 |  |
|  | SVM | 0.6000 | 0.0000 | 0.0000 | 0.0000 | 0.0000 |  |
| WPLI_Alpha_CC_R | DT | 0.5333 | 0.4000 | 0.3333 | 0.0000 | 0.5000 |  |
|  | KNN | 0.6667 | 0.5714 | 0.6667 | 0.3243 | 0.6111 |  |
|  | LR | 0.6000 | 0.0000 | 0.0000 | 0.0000 | 0.0000 |  |
|  | RF | 0.5333 | 0.4000 | 0.3333 | 0.0000 | 0.4074 |  |
|  | SVM | 0.6000 | 0.0000 | 0.0000 | 0.0000 | 0.0000 |  |
| WPLI_Alpha_CC_T | DT | 0.6000 | 0.5000 | 0.5000 | 0.1667 | 0.5833 |  |
|  | KNN | 0.8000 | 1.0000 | 0.5000 | 0.5455 | 0.5556 |  |
|  | LR | 0.6000 | 0.0000 | 0.0000 | 0.0000 | 0.0000 |  |
|  | RF | 0.6000 | 0.5000 | 0.5000 | 0.1667 | 0.5000 |  |
|  | SVM | 0.6000 | 0.0000 | 0.0000 | 0.0000 | 0.0000 |  |
| WPLI_Beta_Bet_D | DT | 0.7333 | 0.6250 | 0.8333 | 0.4737 | 0.7500 |  |
|  | KNN | 0.4000 | 0.2857 | 0.3333 | -0.2162 | 0.2593 |  |
|  | LR | 0.6000 | 0.0000 | 0.0000 | 0.0000 | 0.0000 |  |
|  | RF | 0.7333 | 0.6250 | 0.8333 | 0.4737 | 0.5741 |  |
|  | SVM | 0.6000 | 0.0000 | 0.0000 | 0.0000 | 0.0000 |  |
| WPLI_Beta_Bet_N | DT | 0.4667 | 0.2500 | 0.1667 | -0.1765 | 0.4167 |  |
|  | KNN | 0.6000 | 0.5000 | 0.5000 | 0.1667 | 0.5556 |  |
|  | LR | 0.6000 | 0.0000 | 0.0000 | 0.0000 | 0.0000 |  |
|  | RF | 0.4667 | 0.2500 | 0.1667 | -0.1765 | 0.5000 |  |
|  | SVM | 0.6000 | 0.0000 | 0.0000 | 0.0000 | 0.0000 |  |
| WPLI_Beta_Bet_R | DT | 0.5333 | 0.3333 | 0.1667 | -0.0606 | 0.5370 |  |
|  | KNN | 0.7333 | 0.7500 | 0.5000 | 0.4118 | 0.6944 |  |
|  | LR | 0.6000 | 0.0000 | 0.0000 | 0.0000 | 0.0000 |  |
|  | RF | 0.6000 | 0.5000 | 0.3333 | 0.1176 | 0.5741 |  |
|  | SVM | 0.6000 | 0.0000 | 0.0000 | 0.0000 | 0.0000 |  |
| WPLI_Beta_Bet_T | DT | 0.6000 | 0.5000 | 0.3333 | 0.1176 | 0.5556 |  |
|  | KNN | 0.2000 | 0.0000 | 0.0000 | -0.6667 | 0.1296 |  |
|  | LR | 0.6000 | 0.0000 | 0.0000 | 0.0000 | 0.0000 |  |
|  | RF | 0.6000 | 0.5000 | 0.3333 | 0.1176 | 0.3333 |  |
|  | SVM | 0.6000 | 0.0000 | 0.0000 | 0.0000 | 0.0000 |  |
| WPLI_Beta_De_D | DT | 0.6000 | 0.5000 | 0.5000 | 0.1667 | 0.5833 |  |
|  | KNN | 0.3333 | 0.0000 | 0.0000 | -0.4706 | 0.1481 |  |
|  | LR | 0.4667 | 0.0000 | 0.0000 | -0.2500 | 0.3333 |  |
|  | RF | 0.6000 | 0.5000 | 0.5000 | 0.1667 | 0.3704 |  |
|  | SVM | 0.4667 | 0.0000 | 0.0000 | -0.2500 | 0.1296 |  |
| WPLI_Beta_De_N | DT | 0.7333 | 0.7500 | 0.5000 | 0.4118 | 0.6944 |  |
|  | KNN | 0.8000 | 1.0000 | 0.5000 | 0.5455 | 0.5556 |  |
|  | LR | 0.6667 | 0.6000 | 0.5000 | 0.2857 | 0.6296 |  |
|  | RF | 0.7333 | 0.7500 | 0.5000 | 0.4118 | 0.5000 |  |
|  | SVM | 0.6667 | 0.6000 | 0.5000 | 0.2857 | 0.3889 |  |
| WPLI_Beta_De_R | DT | 0.4667 | 0.2500 | 0.1667 | -0.1765 | 0.4167 |  |
|  | KNN | 0.6667 | 0.6667 | 0.3333 | 0.2424 | 0.4259 |  |
|  | LR | 0.6667 | 0.6667 | 0.3333 | 0.2424 | 0.5000 |  |
|  | RF | 0.4667 | 0.2500 | 0.1667 | -0.1765 | 0.3148 |  |
|  | SVM | 0.5333 | 0.0000 | 0.0000 | -0.1290 | 0.0000 |  |
| WPLI_Beta_De_T | DT | 0.4667 | 0.2500 | 0.1667 | -0.1765 | 0.4167 |  |
|  | KNN | 0.6000 | 0.5000 | 0.1667 | 0.0625 | 0.5278 |  |
|  | LR | 0.6667 | 0.6667 | 0.3333 | 0.2424 | 0.5926 |  |
|  | RF | 0.4667 | 0.2500 | 0.1667 | -0.1765 | 0.3333 |  |
|  | SVM | 0.6000 | 0.5000 | 0.1667 | 0.0625 | 0.1111 |  |
| WPLI_Beta_Clo_D | DT | 0.4000 | 0.2000 | 0.1667 | -0.2857 | 0.3611 |  |
|  | KNN | 0.2000 | 0.0000 | 0.0000 | -0.6667 | 0.0833 |  |
|  | LR | 0.4667 | 0.0000 | 0.0000 | -0.2500 | 0.3889 |  |
|  | RF | 0.4000 | 0.2000 | 0.1667 | -0.2857 | 0.1852 |  |
|  | SVM | 0.4000 | 0.0000 | 0.0000 | -0.3636 | 0.3148 |  |
| WPLI_Beta_Clo_N | DT | 0.7333 | 0.6667 | 0.6667 | 0.4444 | 0.7222 |  |
|  | KNN | 0.8667 | 1.0000 | 0.6667 | 0.7059 | 0.7037 |  |
|  | LR | 0.7333 | 0.6667 | 0.6667 | 0.4444 | 0.6481 |  |
|  | RF | 0.7333 | 0.6667 | 0.6667 | 0.4444 | 0.7037 |  |
|  | SVM | 0.6667 | 0.6000 | 0.5000 | 0.2857 | 0.0000 |  |
| WPLI_Beta_Clo_R | DT | 0.4000 | 0.2000 | 0.1667 | -0.2857 | 0.3611 |  |
|  | KNN | 0.4667 | 0.0000 | 0.0000 | -0.2500 | 0.1944 |  |
|  | LR | 0.3333 | 0.0000 | 0.0000 | -0.4706 | 0.3519 |  |
|  | RF | 0.4000 | 0.2000 | 0.1667 | -0.2857 | 0.1111 |  |
|  | SVM | 0.3333 | 0.0000 | 0.0000 | -0.4706 | 0.5185 |  |
| WPLI_Beta_Clo_T | DT | 0.6000 | 0.5000 | 0.5000 | 0.1667 | 0.5833 |  |
|  | KNN | 0.7333 | 0.7500 | 0.5000 | 0.4118 | 0.5833 |  |
|  | LR | 0.7333 | 0.6667 | 0.6667 | 0.4444 | 0.7037 |  |
|  | RF | 0.6000 | 0.5000 | 0.5000 | 0.1667 | 0.5000 |  |
|  | SVM | 0.6667 | 0.6000 | 0.5000 | 0.2857 | 0.4259 |  |
| WPLI_Beta_CC_D | DT | 0.6667 | 0.5714 | 0.6667 | 0.3243 | 0.6667 |  |
|  | KNN | 0.4000 | 0.2857 | 0.3333 | -0.2162 | 0.4815 |  |
|  | LR | 0.6000 | 0.0000 | 0.0000 | 0.0000 | 0.0000 |  |
|  | RF | 0.6667 | 0.5714 | 0.6667 | 0.3243 | 0.5556 |  |
|  | SVM | 0.6000 | 0.0000 | 0.0000 | 0.0000 | 0.0000 |  |
| WPLI_Beta_CC_N | DT | 0.6667 | 0.5714 | 0.6667 | 0.3243 | 0.6667 |  |
|  | KNN | 0.8000 | 0.8000 | 0.6667 | 0.5714 | 0.6481 |  |
|  | LR | 0.6000 | 0.0000 | 0.0000 | 0.0000 | 0.0000 |  |
|  | RF | 0.6667 | 0.5714 | 0.6667 | 0.3243 | 0.6481 |  |
|  | SVM | 0.6000 | 0.0000 | 0.0000 | 0.0000 | 0.0000 |  |
| WPLI_Beta_CC_R | DT | 0.6667 | 0.6000 | 0.5000 | 0.2857 | 0.6389 |  |
|  | KNN | 0.4667 | 0.0000 | 0.0000 | -0.2500 | 0.1944 |  |
|  | LR | 0.6000 | 0.0000 | 0.0000 | 0.0000 | 0.0000 |  |
|  | RF | 0.6667 | 0.6000 | 0.5000 | 0.2857 | 0.5556 |  |
|  | SVM | 0.6000 | 0.0000 | 0.0000 | 0.0000 | 0.0000 |  |
| WPLI_Beta_CC_T | DT | 0.4667 | 0.2500 | 0.1667 | -0.1765 | 0.4167 |  |
|  | KNN | 0.6667 | 0.6000 | 0.5000 | 0.2857 | 0.6111 |  |
|  | LR | 0.6000 | 0.0000 | 0.0000 | 0.0000 | 0.0000 |  |
|  | RF | 0.4667 | 0.2500 | 0.1667 | -0.1765 | 0.4815 |  |
|  | SVM | 0.6000 | 0.0000 | 0.0000 | 0.0000 | 0.0000 |  |
| WPLI_Delta_Bet_D | DT | 0.6000 | 0.5000 | 0.5000 | 0.1667 | 0.5833 |  |
|  | KNN | 0.4000 | 0.0000 | 0.0000 | -0.3636 | 0.5000 |  |
|  | LR | 0.6000 | 0.0000 | 0.0000 | 0.0000 | 0.0000 |  |
|  | RF | 0.6000 | 0.5000 | 0.5000 | 0.1667 | 0.5741 |  |
|  | SVM | 0.6000 | 0.0000 | 0.0000 | 0.0000 | 0.0000 |  |
| WPLI_Delta_Bet_N | DT | 0.4667 | 0.2500 | 0.1667 | -0.1765 | 0.4167 |  |
|  | KNN | 0.7333 | 0.7500 | 0.5000 | 0.4118 | 0.5556 |  |
|  | LR | 0.6000 | 0.0000 | 0.0000 | 0.0000 | 0.0000 |  |
|  | RF | 0.4667 | 0.2500 | 0.1667 | -0.1765 | 0.4815 |  |
|  | SVM | 0.6000 | 0.0000 | 0.0000 | 0.0000 | 0.1667 |  |
| WPLI_Delta_Bet_R | DT | 0.2000 | 0.0000 | 0.0000 | -0.6667 | 0.1944 |  |
|  | KNN | 0.4667 | 0.0000 | 0.0000 | -0.2500 | 0.2500 |  |
|  | LR | 0.6000 | 0.0000 | 0.0000 | 0.0000 | 0.0000 |  |
|  | RF | 0.2667 | 0.1429 | 0.1667 | -0.4865 | 0.2500 |  |
|  | SVM | 0.6000 | 0.0000 | 0.0000 | 0.0000 | 0.1667 |  |
| WPLI_Delta_Bet_T | DT | 0.3333 | 0.0000 | 0.0000 | -0.4706 | 0.2222 |  |
|  | KNN | 0.2667 | 0.0000 | 0.0000 | -0.5714 | 0.1111 |  |
|  | LR | 0.6000 | 0.0000 | 0.0000 | 0.0000 | 0.0000 |  |
|  | RF | 0.3333 | 0.0000 | 0.0000 | -0.4706 | 0.1759 |  |
|  | SVM | 0.6000 | 0.0000 | 0.0000 | 0.0000 | 0.1667 |  |
| WPLI_Delta_De_D | DT | 0.2667 | 0.1429 | 0.1667 | -0.4865 | 0.2500 |  |
|  | KNN | 0.4667 | 0.0000 | 0.0000 | -0.2500 | 0.1296 |  |
|  | LR | 0.5333 | 0.0000 | 0.0000 | -0.1290 | 0.0000 |  |
|  | RF | 0.2667 | 0.1429 | 0.1667 | -0.4865 | 0.1296 |  |
|  | SVM | 0.5333 | 0.0000 | 0.0000 | -0.1290 | 0.3889 |  |
| WPLI_Delta_De_N | DT | 0.6667 | 0.5714 | 0.6667 | 0.3243 | 0.6667 |  |
|  | KNN | 0.3333 | 0.0000 | 0.0000 | -0.4706 | 0.2778 |  |
|  | LR | 0.7333 | 0.7500 | 0.5000 | 0.4118 | 0.5741 |  |
|  | RF | 0.6667 | 0.5714 | 0.6667 | 0.3243 | 0.6759 |  |
|  | SVM | 0.6000 | 0.5000 | 0.1667 | 0.0625 | 0.0926 |  |
| WPLI_Delta_De_R | DT | 0.4667 | 0.3333 | 0.3333 | -0.1111 | 0.4444 |  |
|  | KNN | 0.6000 | 0.5000 | 0.6667 | 0.2105 | 0.5185 |  |
|  | LR | 0.5333 | 0.0000 | 0.0000 | -0.1290 | 0.0000 |  |
|  | RF | 0.4667 | 0.3333 | 0.3333 | -0.1111 | 0.4352 |  |
|  | SVM | 0.6000 | 0.0000 | 0.0000 | 0.0000 | 0.0000 |  |
| WPLI_Delta_De_T | DT | 0.6667 | 0.6667 | 0.3333 | 0.2424 | 0.6111 |  |
|  | KNN | 0.4000 | 0.0000 | 0.0000 | -0.3636 | 0.3519 |  |
|  | LR | 0.6000 | 0.5000 | 0.5000 | 0.1667 | 0.5556 |  |
|  | RF | 0.6667 | 0.6667 | 0.3333 | 0.2424 | 0.5000 |  |
|  | SVM | 0.4000 | 0.0000 | 0.0000 | -0.3636 | 0.0000 |  |
| WPLI_Delta_Clo_D | DT | 0.6000 | 0.5000 | 0.5000 | 0.1667 | 0.5833 |  |
|  | KNN | 0.4667 | 0.0000 | 0.0000 | -0.2500 | 0.5093 |  |
|  | LR | 0.4667 | 0.0000 | 0.0000 | -0.2500 | 0.3519 |  |
|  | RF | 0.6000 | 0.5000 | 0.5000 | 0.1667 | 0.4630 |  |
|  | SVM | 0.2667 | 0.0000 | 0.0000 | -0.5714 | 0.4630 |  |
| WPLI_Delta_Clo_N | DT | 0.5333 | 0.4000 | 0.3333 | 0.0000 | 0.5000 |  |
|  | KNN | 0.4667 | 0.2500 | 0.1667 | -0.1765 | 0.4352 |  |
|  | LR | 0.4667 | 0.2500 | 0.1667 | -0.1765 | 0.4815 |  |
|  | RF | 0.5333 | 0.4000 | 0.3333 | 0.0000 | 0.4167 |  |
|  | SVM | 0.4000 | 0.0000 | 0.0000 | -0.3636 | 0.0185 |  |
| WPLI_Delta_Clo_R | DT | 0.4667 | 0.3333 | 0.3333 | -0.1111 | 0.4444 |  |
|  | KNN | 0.5333 | 0.4000 | 0.3333 | 0.0000 | 0.4259 |  |
|  | LR | 0.4667 | 0.0000 | 0.0000 | -0.2500 | 0.3889 |  |
|  | RF | 0.4667 | 0.3333 | 0.3333 | -0.1111 | 0.4259 |  |
|  | SVM | 0.4667 | 0.0000 | 0.0000 | -0.2500 | 0.7778 |  |
| WPLI_Delta_Clo_T | DT | 0.6000 | 0.5000 | 0.6667 | 0.2105 | 0.6111 |  |
|  | KNN | 0.6667 | 0.6667 | 0.3333 | 0.2424 | 0.5370 |  |
|  | LR | 0.6000 | 0.5000 | 0.6667 | 0.2105 | 0.5926 |  |
|  | RF | 0.6000 | 0.5000 | 0.6667 | 0.2105 | 0.6759 |  |
|  | SVM | 0.5333 | 0.4286 | 0.5000 | 0.0541 | 0.1296 |  |
| WPLI_Delta_CC_D | DT | 0.8667 | 0.8333 | 0.8333 | 0.7222 | 0.8611 |  |
|  | KNN | 0.3333 | 0.0000 | 0.0000 | -0.4706 | 0.4259 |  |
|  | LR | 0.6000 | 0.0000 | 0.0000 | 0.0000 | 0.0000 |  |
|  | RF | 0.8667 | 0.8333 | 0.8333 | 0.7222 | 0.8426 |  |
|  | SVM | 0.6000 | 0.0000 | 0.0000 | 0.0000 | 0.0000 |  |
| WPLI_Delta_CC_N | DT | 0.5333 | 0.4000 | 0.3333 | 0.0000 | 0.5000 |  |
|  | KNN | 0.3333 | 0.0000 | 0.0000 | -0.4706 | 0.3426 |  |
|  | LR | 0.6000 | 0.0000 | 0.0000 | 0.0000 | 0.0000 |  |
|  | RF | 0.5333 | 0.4000 | 0.3333 | 0.0000 | 0.3519 |  |
|  | SVM | 0.6000 | 0.0000 | 0.0000 | 0.0000 | 0.0000 |  |
| WPLI_Delta_CC_R | DT | 0.6000 | 0.5000 | 0.3333 | 0.1176 | 0.5556 |  |
|  | KNN | 0.5333 | 0.3333 | 0.1667 | -0.0606 | 0.3796 |  |
|  | LR | 0.6000 | 0.0000 | 0.0000 | 0.0000 | 0.0000 |  |
|  | RF | 0.6000 | 0.5000 | 0.3333 | 0.1176 | 0.4815 |  |
|  | SVM | 0.6000 | 0.0000 | 0.0000 | 0.0000 | 0.1667 |  |
| WPLI_Delta_CC_T | DT | 0.2667 | 0.0000 | 0.0000 | -0.5714 | 0.2222 |  |
|  | KNN | 0.4667 | 0.2500 | 0.1667 | -0.1765 | 0.3241 |  |
|  | LR | 0.6000 | 0.0000 | 0.0000 | 0.0000 | 0.0000 |  |
|  | RF | 0.2667 | 0.0000 | 0.0000 | -0.5714 | 0.1667 |  |
|  | SVM | 0.6000 | 0.0000 | 0.0000 | 0.0000 | 0.0000 |  |
| WPLI_Gamma_Bet_D | DT | 0.5333 | 0.4000 | 0.3333 | 0.0000 | 0.5185 |  |
|  | KNN | 0.4000 | 0.0000 | 0.0000 | -0.3636 | 0.2593 |  |
|  | LR | 0.6000 | 0.0000 | 0.0000 | 0.0000 | 0.0000 |  |
|  | RF | 0.4667 | 0.3333 | 0.3333 | -0.1111 | 0.5000 |  |
|  | SVM | 0.6000 | 0.0000 | 0.0000 | 0.0000 | 0.1667 |  |
| WPLI_Gamma_Bet_N | DT | 0.7333 | 0.7500 | 0.5000 | 0.4118 | 0.6944 |  |
|  | KNN | 0.6667 | 0.5714 | 0.6667 | 0.3243 | 0.5648 |  |
|  | LR | 0.6000 | 0.0000 | 0.0000 | 0.0000 | 0.0000 |  |
|  | RF | 0.7333 | 0.7500 | 0.5000 | 0.4118 | 0.6481 |  |
|  | SVM | 0.6000 | 0.0000 | 0.0000 | 0.0000 | 0.1667 |  |
| WPLI_Gamma_Bet_R | DT | 0.1333 | 0.0000 | 0.0000 | -0.7568 | 0.1296 |  |
|  | KNN | 0.4000 | 0.2000 | 0.1667 | -0.2857 | 0.3333 |  |
|  | LR | 0.6000 | 0.0000 | 0.0000 | 0.0000 | 0.0000 |  |
|  | RF | 0.1333 | 0.0000 | 0.0000 | -0.7568 | 0.1481 |  |
|  | SVM | 0.6000 | 0.0000 | 0.0000 | 0.0000 | 0.1667 |  |
| WPLI_Gamma_Bet_T | DT | 0.2667 | 0.0000 | 0.0000 | -0.5714 | 0.2222 |  |
|  | KNN | 0.4000 | 0.0000 | 0.0000 | -0.3636 | 0.2593 |  |
|  | LR | 0.6000 | 0.0000 | 0.0000 | 0.0000 | 0.0000 |  |
|  | RF | 0.2667 | 0.0000 | 0.0000 | -0.5714 | 0.0741 |  |
|  | SVM | 0.6000 | 0.0000 | 0.0000 | 0.0000 | 0.3333 |  |
| WPLI_Gamma_De_D | DT | 0.6667 | 0.6000 | 0.5000 | 0.2857 | 0.6389 |  |
|  | KNN | 0.6000 | 0.0000 | 0.0000 | 0.0000 | 0.3519 |  |
|  | LR | 0.6000 | 0.0000 | 0.0000 | 0.0000 | 0.0000 |  |
|  | RF | 0.6667 | 0.6000 | 0.5000 | 0.2857 | 0.5278 |  |
|  | SVM | 0.6000 | 0.0000 | 0.0000 | 0.0000 | 0.2222 |  |
| WPLI_Gamma_De_N | DT | 0.2667 | 0.0000 | 0.0000 | -0.5714 | 0.2222 |  |
|  | KNN | 0.5333 | 0.0000 | 0.0000 | -0.1290 | 0.1296 |  |
|  | LR | 0.5333 | 0.0000 | 0.0000 | -0.1290 | 0.0000 |  |
|  | RF | 0.2667 | 0.0000 | 0.0000 | -0.5714 | 0.0000 |  |
|  | SVM | 0.5333 | 0.0000 | 0.0000 | -0.1290 | 0.3333 |  |
| WPLI_Gamma_De_R | DT | 0.6667 | 0.5714 | 0.6667 | 0.3243 | 0.6667 |  |
|  | KNN | 0.5333 | 0.0000 | 0.0000 | -0.1290 | 0.2222 |  |
|  | LR | 0.6000 | 0.0000 | 0.0000 | 0.0000 | 0.0000 |  |
|  | RF | 0.6667 | 0.5714 | 0.6667 | 0.3243 | 0.5556 |  |
|  | SVM | 0.6000 | 0.0000 | 0.0000 | 0.0000 | 0.1111 |  |
| WPLI_Gamma_De_T | DT | 0.5333 | 0.4286 | 0.5000 | 0.0541 | 0.5278 |  |
|  | KNN | 0.5333 | 0.0000 | 0.0000 | -0.1290 | 0.3796 |  |
|  | LR | 0.6000 | 0.0000 | 0.0000 | 0.0000 | 0.0000 |  |
|  | RF | 0.5333 | 0.4286 | 0.5000 | 0.0541 | 0.4722 |  |
|  | SVM | 0.6000 | 0.0000 | 0.0000 | 0.0000 | 0.6296 |  |
| WPLI_Gamma_Clo_D | DT | 0.4000 | 0.0000 | 0.0000 | -0.3636 | 0.3333 |  |
|  | KNN | 0.6000 | 0.0000 | 0.0000 | 0.0000 | 0.0000 |  |
|  | LR | 0.5333 | 0.0000 | 0.0000 | -0.1290 | 0.0000 |  |
|  | RF | 0.4000 | 0.0000 | 0.0000 | -0.3636 | 0.1111 |  |
|  | SVM | 0.6000 | 0.0000 | 0.0000 | 0.0000 | 0.0000 |  |
| WPLI_Gamma_Clo_N | DT | 0.4667 | 0.2500 | 0.1667 | -0.1765 | 0.4167 |  |
|  | KNN | 0.4667 | 0.0000 | 0.0000 | -0.2500 | 0.3611 |  |
|  | LR | 0.6000 | 0.0000 | 0.0000 | 0.0000 | 0.0000 |  |
|  | RF | 0.4667 | 0.2500 | 0.1667 | -0.1765 | 0.2130 |  |
|  | SVM | 0.6000 | 0.0000 | 0.0000 | 0.0000 | 0.3889 |  |
| WPLI_Gamma_Clo_R | DT | 0.5333 | 0.4286 | 0.5000 | 0.0541 | 0.5278 |  |
|  | KNN | 0.4667 | 0.0000 | 0.0000 | -0.2500 | 0.3333 |  |
|  | LR | 0.4667 | 0.0000 | 0.0000 | -0.2500 | 0.3148 |  |
|  | RF | 0.5333 | 0.4286 | 0.5000 | 0.0541 | 0.5093 |  |
|  | SVM | 0.3333 | 0.0000 | 0.0000 | -0.4706 | 0.2407 |  |
| WPLI_Gamma_Clo_T | DT | 0.6667 | 0.6667 | 0.3333 | 0.2424 | 0.6111 |  |
|  | KNN | 0.6000 | 0.0000 | 0.0000 | 0.0000 | 0.4259 |  |
|  | LR | 0.5333 | 0.0000 | 0.0000 | -0.1290 | 0.0000 |  |
|  | RF | 0.6667 | 0.6667 | 0.3333 | 0.2424 | 0.5000 |  |
|  | SVM | 0.6000 | 0.0000 | 0.0000 | 0.0000 | 0.0185 |  |
| WPLI_Gamma_CC_D | DT | 0.4667 | 0.3333 | 0.3333 | -0.1111 | 0.4444 |  |
|  | KNN | 0.6667 | 0.5714 | 0.6667 | 0.3243 | 0.5370 |  |
|  | LR | 0.6000 | 0.0000 | 0.0000 | 0.0000 | 0.0000 |  |
|  | RF | 0.4667 | 0.3333 | 0.3333 | -0.1111 | 0.4444 |  |
|  | SVM | 0.6000 | 0.0000 | 0.0000 | 0.0000 | 0.0000 |  |
| WPLI_Gamma_CC_N | DT | 0.4000 | 0.2857 | 0.3333 | -0.2162 | 0.3889 |  |
|  | KNN | 0.5333 | 0.0000 | 0.0000 | -0.1290 | 0.4074 |  |
|  | LR | 0.6000 | 0.0000 | 0.0000 | 0.0000 | 0.0000 |  |
|  | RF | 0.4000 | 0.2857 | 0.3333 | -0.2162 | 0.3148 |  |
|  | SVM | 0.6000 | 0.0000 | 0.0000 | 0.0000 | 0.1667 |  |
| WPLI_Gamma_CC_R | DT | 0.4000 | 0.2857 | 0.3333 | -0.2162 | 0.3889 |  |
|  | KNN | 0.4667 | 0.0000 | 0.0000 | -0.2500 | 0.1759 |  |
|  | LR | 0.6000 | 0.0000 | 0.0000 | 0.0000 | 0.0000 |  |
|  | RF | 0.4000 | 0.2857 | 0.3333 | -0.2162 | 0.2500 |  |
|  | SVM | 0.6000 | 0.0000 | 0.0000 | 0.0000 | 0.1667 |  |
| WPLI_Gamma_CC_T | DT | 0.3333 | 0.1667 | 0.1667 | -0.3889 | 0.3056 |  |
|  | KNN | 0.4000 | 0.0000 | 0.0000 | -0.3636 | 0.1111 |  |
|  | LR | 0.6000 | 0.0000 | 0.0000 | 0.0000 | 0.0000 |  |
|  | RF | 0.3333 | 0.1667 | 0.1667 | -0.3889 | 0.2685 |  |
|  | SVM | 0.6000 | 0.0000 | 0.0000 | 0.0000 | 0.0000 |  |
| WPLI_Theta_Bet_D | DT | 0.2000 | 0.0000 | 0.0000 | -0.6667 | 0.1667 |  |
|  | KNN | 0.4667 | 0.2500 | 0.1667 | -0.1765 | 0.4444 |  |
|  | LR | 0.6000 | 0.0000 | 0.0000 | 0.0000 | 0.0000 |  |
|  | RF | 0.2000 | 0.0000 | 0.0000 | -0.6667 | 0.2593 |  |
|  | SVM | 0.6000 | 0.0000 | 0.0000 | 0.0000 | 0.1667 |  |
| WPLI_Theta_Bet_N | DT | 0.5333 | 0.4000 | 0.3333 | 0.0000 | 0.4630 |  |
|  | KNN | 0.4000 | 0.0000 | 0.0000 | -0.3636 | 0.4074 |  |
|  | LR | 0.6000 | 0.0000 | 0.0000 | 0.0000 | 0.0000 |  |
|  | RF | 0.5333 | 0.4000 | 0.3333 | 0.0000 | 0.3981 |  |
|  | SVM | 0.6000 | 0.0000 | 0.0000 | 0.0000 | 0.1667 |  |
| WPLI_Theta_Bet_R | DT | 0.4000 | 0.2857 | 0.3333 | -0.2162 | 0.3889 |  |
|  | KNN | 0.6000 | 0.5000 | 0.3333 | 0.1176 | 0.3889 |  |
|  | LR | 0.6000 | 0.0000 | 0.0000 | 0.0000 | 0.0000 |  |
|  | RF | 0.4000 | 0.2857 | 0.3333 | -0.2162 | 0.3333 |  |
|  | SVM | 0.6000 | 0.0000 | 0.0000 | 0.0000 | 0.0000 |  |
| WPLI_Theta_Bet_T | DT | 0.4000 | 0.2857 | 0.3333 | -0.2162 | 0.3889 |  |
|  | KNN | 0.4000 | 0.0000 | 0.0000 | -0.3636 | 0.2778 |  |
|  | LR | 0.6000 | 0.0000 | 0.0000 | 0.0000 | 0.0000 |  |
|  | RF | 0.4000 | 0.2857 | 0.3333 | -0.2162 | 0.2593 |  |
|  | SVM | 0.6000 | 0.0000 | 0.0000 | 0.0000 | 0.1667 |  |
| WPLI_Theta_De_D | DT | 0.4667 | 0.3333 | 0.3333 | -0.1111 | 0.4444 |  |
|  | KNN | 0.4000 | 0.0000 | 0.0000 | -0.3636 | 0.3889 |  |
|  | LR | 0.6000 | 0.0000 | 0.0000 | 0.0000 | 0.0000 |  |
|  | RF | 0.4667 | 0.3333 | 0.3333 | -0.1111 | 0.4537 |  |
|  | SVM | 0.6000 | 0.0000 | 0.0000 | 0.0000 | 0.2778 |  |
| WPLI_Theta_De_N | DT | 0.7333 | 0.6667 | 0.6667 | 0.4444 | 0.7222 |  |
|  | KNN | 0.4000 | 0.0000 | 0.0000 | -0.3636 | 0.3056 |  |
|  | LR | 0.5333 | 0.3333 | 0.1667 | -0.0606 | 0.4815 |  |
|  | RF | 0.7333 | 0.6667 | 0.6667 | 0.4444 | 0.7778 |  |
|  | SVM | 0.6000 | 0.0000 | 0.0000 | 0.0000 | 0.1667 |  |
| WPLI_Theta_De_R | DT | 0.2667 | 0.2222 | 0.3333 | -0.4103 | 0.2778 |  |
|  | KNN | 0.2667 | 0.0000 | 0.0000 | -0.5714 | 0.1481 |  |
|  | LR | 0.4667 | 0.0000 | 0.0000 | -0.2500 | 0.3519 |  |
|  | RF | 0.2667 | 0.2222 | 0.3333 | -0.4103 | 0.1296 |  |
|  | SVM | 0.4667 | 0.0000 | 0.0000 | -0.2500 | 0.1111 |  |
| WPLI_Theta_De_T | DT | 0.4667 | 0.3333 | 0.3333 | -0.1111 | 0.4444 |  |
|  | KNN | 0.7333 | 0.6000 | 1.0000 | 0.5000 | 0.5556 |  |
|  | LR | 0.6667 | 0.6667 | 0.3333 | 0.2424 | 0.6296 |  |
|  | RF | 0.4667 | 0.3333 | 0.3333 | -0.1111 | 0.5926 |  |
|  | SVM | 0.4667 | 0.0000 | 0.0000 | -0.2500 | 0.1852 |  |
| WPLI_Theta_Clo_D | DT | 0.5333 | 0.4000 | 0.3333 | 0.0000 | 0.5000 |  |
|  | KNN | 0.4667 | 0.0000 | 0.0000 | -0.2500 | 0.1944 |  |
|  | LR | 0.5333 | 0.0000 | 0.0000 | -0.1290 | 0.0000 |  |
|  | RF | 0.5333 | 0.4000 | 0.3333 | 0.0000 | 0.2593 |  |
|  | SVM | 0.6000 | 0.0000 | 0.0000 | 0.0000 | 0.0741 |  |
| WPLI_Theta_Clo_N | DT | 0.6000 | 0.5000 | 0.5000 | 0.1667 | 0.5833 |  |
|  | KNN | 0.4667 | 0.2500 | 0.1667 | -0.1765 | 0.4630 |  |
|  | LR | 0.5333 | 0.4000 | 0.3333 | 0.0000 | 0.5370 |  |
|  | RF | 0.6000 | 0.5000 | 0.5000 | 0.1667 | 0.6111 |  |
|  | SVM | 0.5333 | 0.4000 | 0.3333 | 0.0000 | 0.1852 |  |
| WPLI_Theta_Clo_R | DT | 0.5333 | 0.3333 | 0.1667 | -0.0606 | 0.4722 |  |
|  | KNN | 0.1333 | 0.0000 | 0.0000 | -0.7568 | 0.1111 |  |
|  | LR | 0.4667 | 0.0000 | 0.0000 | -0.2500 | 0.2222 |  |
|  | RF | 0.5333 | 0.3333 | 0.1667 | -0.0606 | 0.3241 |  |
|  | SVM | 0.5333 | 0.0000 | 0.0000 | -0.1290 | 0.2778 |  |
| WPLI_Theta_Clo_T | DT | 0.6667 | 0.5714 | 0.6667 | 0.3243 | 0.6667 |  |
|  | KNN | 0.8000 | 0.6667 | 1.0000 | 0.6154 | 0.7778 |  |
|  | LR | 0.8000 | 0.7143 | 0.8333 | 0.5946 | 0.7778 |  |
|  | RF | 0.6667 | 0.5714 | 0.6667 | 0.3243 | 0.7500 |  |
|  | SVM | 0.8667 | 0.7500 | 1.0000 | 0.7368 | 0.7778 |  |
| WPLI_Theta_CC_D | DT | 0.3333 | 0.0000 | 0.0000 | -0.4706 | 0.2778 |  |
|  | KNN | 0.5333 | 0.0000 | 0.0000 | -0.1290 | 0.2870 |  |
|  | LR | 0.6000 | 0.0000 | 0.0000 | 0.0000 | 0.0000 |  |
|  | RF | 0.3333 | 0.0000 | 0.0000 | -0.4706 | 0.2130 |  |
|  | SVM | 0.6000 | 0.0000 | 0.0000 | 0.0000 | 0.0000 |  |
| WPLI_Theta_CC_N | DT | 0.3333 | 0.1667 | 0.1667 | -0.3889 | 0.3056 |  |
|  | KNN | 0.4000 | 0.0000 | 0.0000 | -0.3636 | 0.1111 |  |
|  | LR | 0.6000 | 0.0000 | 0.0000 | 0.0000 | 0.0000 |  |
|  | RF | 0.3333 | 0.1667 | 0.1667 | -0.3889 | 0.2037 |  |
|  | SVM | 0.6000 | 0.0000 | 0.0000 | 0.0000 | 0.0000 |  |
| WPLI_Theta_CC_R | DT | 0.3333 | 0.1667 | 0.1667 | -0.3889 | 0.3056 |  |
|  | KNN | 0.3333 | 0.0000 | 0.0000 | -0.4706 | 0.1944 |  |
|  | LR | 0.6000 | 0.0000 | 0.0000 | 0.0000 | 0.0000 |  |
|  | RF | 0.3333 | 0.1667 | 0.1667 | -0.3889 | 0.3148 |  |
|  | SVM | 0.6000 | 0.0000 | 0.0000 | 0.0000 | 0.0000 |  |
| WPLI_Theta_CC_T | DT | 0.3333 | 0.0000 | 0.0000 | -0.4706 | 0.2778 |  |
|  | KNN | 0.4000 | 0.0000 | 0.0000 | -0.3636 | 0.2778 |  |
|  | LR | 0.6000 | 0.0000 | 0.0000 | 0.0000 | 0.0000 |  |
|  | RF | 0.3333 | 0.0000 | 0.0000 | -0.4706 | 0.1759 |  |
|  | SVM | 0.6000 | 0.0000 | 0.0000 | 0.0000 | 0.0000 |  |
| WPLI_Band_Bet_D | DT | 0.4667 | 0.3333 | 0.3333 | -0.1111 | 0.4444 |  |
|  | KNN | 0.4667 | 0.0000 | 0.0000 | -0.2500 | 0.4259 |  |
|  | LR | 0.6000 | 0.0000 | 0.0000 | 0.0000 | 0.0000 |  |
|  | RF | 0.4667 | 0.3333 | 0.3333 | -0.1111 | 0.2685 |  |
|  | SVM | 0.6000 | 0.0000 | 0.0000 | 0.0000 | 0.0000 |  |
| WPLI_Band_Bet_N | DT | 0.5333 | 0.4000 | 0.3333 | 0.0000 | 0.5000 |  |
|  | KNN | 0.4667 | 0.0000 | 0.0000 | -0.2500 | 0.4722 |  |
|  | LR | 0.6000 | 0.0000 | 0.0000 | 0.0000 | 0.0000 |  |
|  | RF | 0.5333 | 0.4000 | 0.3333 | 0.0000 | 0.5926 |  |
|  | SVM | 0.6000 | 0.0000 | 0.0000 | 0.0000 | 0.1667 |  |
| WPLI_Band_Bet_R | DT | 0.6667 | 0.6000 | 0.5000 | 0.2857 | 0.6389 |  |
|  | KNN | 0.6667 | 0.5556 | 0.8333 | 0.3590 | 0.5278 |  |
|  | LR | 0.6000 | 0.0000 | 0.0000 | 0.0000 | 0.0000 |  |
|  | RF | 0.6667 | 0.6000 | 0.5000 | 0.2857 | 0.6574 |  |
|  | SVM | 0.6000 | 0.0000 | 0.0000 | 0.0000 | 0.1667 |  |
| WPLI_Band_Bet_T | DT | 0.5333 | 0.3333 | 0.1667 | -0.0606 | 0.4259 |  |
|  | KNN | 0.4667 | 0.0000 | 0.0000 | -0.2500 | 0.3519 |  |
|  | LR | 0.6000 | 0.0000 | 0.0000 | 0.0000 | 0.0000 |  |
|  | RF | 0.4667 | 0.2500 | 0.1667 | -0.1765 | 0.2778 |  |
|  | SVM | 0.6000 | 0.0000 | 0.0000 | 0.0000 | 0.5000 |  |
| WPLI_Band_De_D | DT | 0.4667 | 0.3750 | 0.5000 | -0.0526 | 0.4722 |  |
|  | KNN | 0.6667 | 0.6667 | 0.3333 | 0.2424 | 0.3981 |  |
|  | LR | 0.5333 | 0.0000 | 0.0000 | -0.1290 | 0.4074 |  |
|  | RF | 0.4667 | 0.3750 | 0.5000 | -0.0526 | 0.3704 |  |
|  | SVM | 0.3333 | 0.0000 | 0.0000 | -0.4706 | 0.3333 |  |
| WPLI_Band_De_N | DT | 0.6000 | 0.5000 | 0.5000 | 0.1667 | 0.5833 |  |
|  | KNN | 0.7333 | 1.0000 | 0.3333 | 0.3750 | 0.6111 |  |
|  | LR | 0.6667 | 0.6000 | 0.5000 | 0.2857 | 0.6852 |  |
|  | RF | 0.6000 | 0.5000 | 0.5000 | 0.1667 | 0.5000 |  |
|  | SVM | 0.6667 | 0.6000 | 0.5000 | 0.2857 | 0.0926 |  |
| WPLI_Band_De_R | DT | 0.2667 | 0.1429 | 0.1667 | -0.4865 | 0.2500 |  |
|  | KNN | 0.4000 | 0.2000 | 0.1667 | -0.2857 | 0.4907 |  |
|  | LR | 0.4667 | 0.0000 | 0.0000 | -0.2500 | 0.4259 |  |
|  | RF | 0.2667 | 0.1429 | 0.1667 | -0.4865 | 0.2778 |  |
|  | SVM | 0.4667 | 0.0000 | 0.0000 | -0.2500 | 0.3889 |  |
| WPLI_Band_De_T | DT | 0.7333 | 0.6667 | 0.6667 | 0.4444 | 0.7222 |  |
|  | KNN | 0.7333 | 0.6667 | 0.6667 | 0.4444 | 0.7500 |  |
|  | LR | 0.7333 | 0.7500 | 0.5000 | 0.4118 | 0.7222 |  |
|  | RF | 0.7333 | 0.6667 | 0.6667 | 0.4444 | 0.7685 |  |
|  | SVM | 0.5333 | 0.3333 | 0.1667 | -0.0606 | 0.2593 |  |
| WPLI_Band_Clo_D | DT | 0.6000 | 0.5000 | 0.5000 | 0.1667 | 0.5833 |  |
|  | KNN | 0.4667 | 0.2500 | 0.1667 | -0.1765 | 0.3056 |  |
|  | LR | 0.3333 | 0.0000 | 0.0000 | -0.4706 | 0.4074 |  |
|  | RF | 0.6000 | 0.5000 | 0.5000 | 0.1667 | 0.4537 |  |
|  | SVM | 0.2667 | 0.0000 | 0.0000 | -0.5714 | 0.4630 |  |
| WPLI_Band_Clo_N | DT | 0.4000 | 0.2857 | 0.3333 | -0.2162 | 0.3889 |  |
|  | KNN | 0.6000 | 0.5000 | 0.3333 | 0.1176 | 0.5370 |  |
|  | LR | 0.6667 | 0.5714 | 0.6667 | 0.3243 | 0.6296 |  |
|  | RF | 0.4000 | 0.2857 | 0.3333 | -0.2162 | 0.4444 |  |
|  | SVM | 0.6000 | 0.5000 | 0.6667 | 0.2105 | 0.0000 |  |
| WPLI_Band_Clo_R | DT | 0.1333 | 0.0000 | 0.0000 | -0.7568 | 0.1111 |  |
|  | KNN | 0.4667 | 0.3333 | 0.3333 | -0.1111 | 0.4537 |  |
|  | LR | 0.4667 | 0.0000 | 0.0000 | -0.2500 | 0.4074 |  |
|  | RF | 0.1333 | 0.0000 | 0.0000 | -0.7568 | 0.1296 |  |
|  | SVM | 0.3333 | 0.0000 | 0.0000 | -0.4706 | 0.5556 |  |
| WPLI_Band_Clo_T | DT | 0.6667 | 0.6000 | 0.5000 | 0.2857 | 0.6389 |  |
|  | KNN | 0.4667 | 0.2500 | 0.1667 | -0.1765 | 0.5278 |  |
|  | LR | 0.6667 | 0.5714 | 0.6667 | 0.3243 | 0.6852 |  |
|  | RF | 0.6667 | 0.6000 | 0.5000 | 0.2857 | 0.5926 |  |
|  | SVM | 0.6667 | 0.5714 | 0.6667 | 0.3243 | 0.0741 |  |
| WPLI_Band_CC_D | DT | 0.4667 | 0.3333 | 0.3333 | -0.1111 | 0.4444 |  |
|  | KNN | 0.4667 | 0.3750 | 0.5000 | -0.0526 | 0.3704 |  |
|  | LR | 0.6000 | 0.0000 | 0.0000 | 0.0000 | 0.0000 |  |
|  | RF | 0.4667 | 0.3333 | 0.3333 | -0.1111 | 0.3426 |  |
|  | SVM | 0.6000 | 0.0000 | 0.0000 | 0.0000 | 0.0000 |  |
| WPLI_Band_CC_N | DT | 0.4667 | 0.3333 | 0.3333 | -0.1111 | 0.4444 |  |
|  | KNN | 0.6000 | 0.5000 | 0.3333 | 0.1176 | 0.4907 |  |
|  | LR | 0.6000 | 0.0000 | 0.0000 | 0.0000 | 0.0000 |  |
|  | RF | 0.4667 | 0.3333 | 0.3333 | -0.1111 | 0.4537 |  |
|  | SVM | 0.6000 | 0.0000 | 0.0000 | 0.0000 | 0.0000 |  |
| WPLI_Band_CC_R | DT | 0.4667 | 0.3333 | 0.3333 | -0.1111 | 0.4444 |  |
|  | KNN | 0.6000 | 0.0000 | 0.0000 | 0.0000 | 0.2778 |  |
|  | LR | 0.6000 | 0.0000 | 0.0000 | 0.0000 | 0.0000 |  |
|  | RF | 0.4667 | 0.3333 | 0.3333 | -0.1111 | 0.3148 |  |
|  | SVM | 0.6000 | 0.0000 | 0.0000 | 0.0000 | 0.0000 |  |
| WPLI_Band_CC_T | DT | 0.4667 | 0.3333 | 0.3333 | -0.1111 | 0.4444 |  |
|  | KNN | 0.4667 | 0.3333 | 0.3333 | -0.1111 | 0.4444 |  |
|  | LR | 0.6000 | 0.0000 | 0.0000 | 0.0000 | 0.0000 |  |
|  | RF | 0.4667 | 0.3333 | 0.3333 | -0.1111 | 0.4167 |  |
|  | SVM | 0.6000 | 0.0000 | 0.0000 | 0.0000 | 0.0185 |  |

WPLI: Weighted Phase Lag Index; De: Degree; CC: Clustering Coefficient; Clo: Closeness Centrality; Bet: Betweenness Centrality; PSD: Power Spectral Density; PE: Permutation Entropy; AE: Approximate Entropy; SAE: Sample Entropy; SPE: Spectral Entropy; LZC: Lempel-Ziv Complexity; Band: Full Frequency Band; T: Pure-tone Paradigm; N: Name-calling Paradigm; R: Reversed-word Sequence Paradigm; D: Different-word Sequence Paradigm

Supplementary Table 4. Regression Results Table for All Single Features

| **Feature** | **OLS** | | | | **SVR** | |
| --- | --- | --- | --- | --- | --- | --- |
|  | **R^2^_cls** | **P_cls** | **R^2^_score** | **P_ score** | **R^2^_ score** | **P_ score** |
| LZC_D | 0.2499 | 0.0578 | 0.1268 | 0.1926 | 0.3291 | 0.0254 |
| LZC_N | 0.2392 | 0.0643 | 0.0767 | 0.3177 | 0.1278 | 0.1908 |
| LZC_R | 0.3684 | 0.0164 | 0.1457 | 0.1603 | 0.3126 | 0.0302 |
| LZC_T | 0.1606 | 0.1388 | 0.0326 | 0.5199 | 0.0364 | 0.4958 |
| SPE_D | 0.1872 | 0.1072 | 0.1292 | 0.1883 | 0.1704 | 0.1262 |
| SPE_N | 0.1986 | 0.0959 | 0.1079 | 0.2319 | 0.1687 | 0.1283 |
| SPE_R | 0.3505 | 0.0201 | 0.1745 | 0.1212 | 0.1871 | 0.1073 |
| SPE_T | 0.2235 | 0.0751 | 0.0792 | 0.3097 | 0.2024 | 0.0925 |
| PE_D | 0.0226 | 0.5925 | 0.0006 | 0.9302 | 0.4041 | 0.0109 |
| PE_N | 0.0159 | 0.6544 | 0.0747 | 0.3244 | 0.3114 | 0.0306 |
| PE_R | 0.0483 | 0.4314 | 0.0000 | 0.9834 | 0.3842 | 0.0137 |
| PE_T | 0.0391 | 0.4798 | 0.3674 | 0.0166 | 0.0650 | 0.3593 |
| SAE_D | 0.2392 | 0.0643 | 0.1206 | 0.2048 | 0.3194 | 0.0281 |
| SAE_N | 0.2347 | 0.0673 | 0.0775 | 0.3152 | 0.1264 | 0.1934 |
| SAE_R | 0.3641 | 0.0172 | 0.1408 | 0.1682 | 0.3055 | 0.0326 |
| SAE_T | 0.1917 | 0.1026 | 0.0493 | 0.4264 | 0.0569 | 0.3921 |
| AE_D | 0.2371 | 0.0657 | 0.1198 | 0.2064 | 0.3169 | 0.0289 |
| AE_N | 0.2333 | 0.0682 | 0.0764 | 0.3188 | 0.1265 | 0.1932 |
| AE_R | 0.3528 | 0.0196 | 0.1341 | 0.1795 | 0.3035 | 0.0333 |
| AE_T | 0.1922 | 0.1021 | 0.0489 | 0.4285 | 0.0688 | 0.3448 |
| PSD_Alpha_D | 0.0105 | 0.7167 | 0.0004 | 0.9408 | 0.0183 | 0.6308 |
| PSD_Alpha_N | 0.0028 | 0.8511 | 0.0062 | 0.7799 | 0.1356 | 0.1769 |
| PSD_Alpha_R | 0.0345 | 0.5078 | 0.0115 | 0.7035 | 0.1505 | 0.1531 |
| PSD_Alpha_T | 0.0764 | 0.3188 | 0.0768 | 0.3174 | 0.0087 | 0.7410 |
| PSD_Beta_D | 0.1416 | 0.1668 | 0.0572 | 0.3907 | 0.0645 | 0.3610 |
| PSD_Beta_N | 0.1567 | 0.1441 | 0.0352 | 0.5029 | 0.0116 | 0.7029 |
| PSD_Beta_R | 0.2160 | 0.0809 | 0.0587 | 0.3842 | 0.3947 | 0.0121 |
| PSD_Beta_T | 0.1875 | 0.1069 | 0.0466 | 0.4396 | 0.0275 | 0.5545 |
| PSD_Delta_D | 0.0900 | 0.2772 | 0.0596 | 0.3806 | 0.2113 | 0.0847 |
| PSD_Delta_N | 0.1104 | 0.2263 | 0.0880 | 0.2830 | 0.3539 | 0.0193 |
| PSD_Delta_R | 0.2536 | 0.0557 | 0.1024 | 0.2448 | 0.1369 | 0.1747 |
| PSD_Delta_T | 0.1849 | 0.1097 | 0.1095 | 0.2284 | 0.1834 | 0.1112 |
| PSD_Gamma_D | 0.1834 | 0.1112 | 0.0819 | 0.3011 | 0.2136 | 0.0829 |
| PSD_Gamma_N | 0.1005 | 0.2497 | 0.0069 | 0.7678 | 0.1213 | 0.2033 |
| PSD_Gamma_R | 0.1660 | 0.1317 | 0.0370 | 0.4925 | 0.4285 | 0.0081 |
| PSD_Gamma_T | 0.0354 | 0.5018 | 0.1260 | 0.1941 | 0.1252 | 0.1957 |
| PSD_Theta_D | 0.0078 | 0.7545 | 0.0142 | 0.6723 | 0.0539 | 0.4049 |
| PSD_Theta_N | 0.0129 | 0.6867 | 0.0004 | 0.9414 | 0.0358 | 0.4996 |
| PSD_Theta_R | 0.0043 | 0.8158 | 0.0013 | 0.8987 | 0.0166 | 0.6468 |
| PSD_Theta_T | 0.0154 | 0.6600 | 0.0065 | 0.7753 | 0.0125 | 0.6915 |
| WPLI_Alpha_Bet_D | 0.0001 | 0.9703 | 0.0166 | 0.6473 | 0.0001 | 0.9732 |
| WPLI_Alpha_Bet_N | 0.1757 | 0.1198 | 0.8577 | 0.0000 | 0.2065 | 0.0888 |
| WPLI_Alpha_Bet_R | 0.6320 | 0.0004 | 0.0109 | 0.7110 | 0.0002 | 0.9578 |
| WPLI_Alpha_Bet_T | 0.1092 | 0.2290 | 0.0261 | 0.5650 | 0.0002 | 0.9573 |
| WPLI_Alpha_De_D | 0.0403 | 0.4729 | 0.0099 | 0.7247 | 0.0000 | 0.9912 |
| WPLI_Alpha_De_N | 0.0101 | 0.7209 | 0.0988 | 0.2539 | 0.0425 | 0.4610 |
| WPLI_Alpha_De_R | 0.0765 | 0.3184 | 0.0044 | 0.8137 | 0.0083 | 0.7469 |
| WPLI_Alpha_De_T | 0.1127 | 0.2211 | 0.2701 | 0.0471 | 0.2006 | 0.0941 |
| WPLI_Alpha_Clo_D | 0.0755 | 0.3216 | 0.0143 | 0.6713 | 0.0061 | 0.7812 |
| WPLI_Alpha_Clo_N | 0.0007 | 0.9235 | 0.0187 | 0.6270 | 0.0022 | 0.8681 |
| WPLI_Alpha_Clo_R | 0.0080 | 0.7508 | 0.0060 | 0.7834 | 0.0417 | 0.4655 |
| WPLI_Alpha_Clo_T | 0.1625 | 0.1363 | 0.1938 | 0.1005 | 0.0001 | 0.9678 |
| WPLI_Alpha_CC_D | 0.4387 | 0.0071 | 0.5158 | 0.0026 | 0.3582 | 0.0184 |
| WPLI_Alpha_CC_N | 0.3495 | 0.0203 | 0.0220 | 0.5980 | 0.0669 | 0.3518 |
| WPLI_Alpha_CC_R | 0.0682 | 0.3472 | 0.0209 | 0.6069 | 0.0002 | 0.9581 |
| WPLI_Alpha_CC_T | 0.1189 | 0.2082 | 0.0196 | 0.6191 | 0.0705 | 0.3390 |
| WPLI_Beta_Bet_D | 0.0359 | 0.4991 | 0.1538 | 0.1483 | 0.2670 | 0.0486 |
| WPLI_Beta_Bet_N | 0.0067 | 0.7715 | 0.1520 | 0.1508 | 0.2408 | 0.0633 |
| WPLI_Beta_Bet_R | 0.0031 | 0.8427 | 0.0497 | 0.4244 | 0.0032 | 0.8419 |
| WPLI_Beta_Bet_T | 0.7465 | 0.0000 | 0.3825 | 0.0140 | 0.3212 | 0.0276 |
| WPLI_Beta_De_D | 0.0291 | 0.5430 | 0.0423 | 0.4621 | 0.2449 | 0.0607 |
| WPLI_Beta_De_N | 0.0954 | 0.2627 | 0.0381 | 0.4854 | 0.0000 | 0.9816 |
| WPLI_Beta_De_R | 0.0001 | 0.9750 | 0.0153 | 0.6600 | 0.0942 | 0.2659 |
| WPLI_Beta_De_T | 0.0976 | 0.2570 | 0.1760 | 0.1196 | 0.0146 | 0.6683 |
| WPLI_Beta_Clo_D | 0.0872 | 0.2854 | 0.0096 | 0.7283 | 0.1705 | 0.1261 |
| WPLI_Beta_Clo_N | 0.0245 | 0.5778 | 0.0710 | 0.3369 | 0.3534 | 0.0194 |
| WPLI_Beta_Clo_R | 0.0884 | 0.2819 | 0.0005 | 0.9395 | 0.1145 | 0.2174 |
| WPLI_Beta_Clo_T | 0.1198 | 0.2063 | 0.1710 | 0.1255 | 0.2506 | 0.0573 |
| WPLI_Beta_CC_D | 0.0239 | 0.5819 | 0.0000 | 0.9840 | 0.0691 | 0.3438 |
| WPLI_Beta_CC_N | 0.4580 | 0.0056 | 0.0121 | 0.6960 | 0.0034 | 0.8362 |
| WPLI_Beta_CC_R | 0.0112 | 0.7069 | 0.0003 | 0.9522 | 0.0321 | 0.5232 |
| WPLI_Beta_CC_T | 0.0033 | 0.8386 | 0.0005 | 0.9373 | 0.0108 | 0.7121 |
| WPLI_Delta_Bet_D | 0.3835 | 0.0138 | 0.0033 | 0.8386 | 0.0201 | 0.6140 |
| WPLI_Delta_Bet_N | 0.0241 | 0.5805 | 0.4787 | 0.0043 | 0.0000 | 0.9920 |
| WPLI_Delta_Bet_R | 0.3794 | 0.0145 | 0.0707 | 0.3381 | 0.2830 | 0.0412 |
| WPLI_Delta_Bet_T | 0.0059 | 0.7861 | 0.2169 | 0.0802 | 0.0032 | 0.8410 |
| WPLI_Delta_De_D | 0.3984 | 0.0116 | 0.0507 | 0.4198 | 0.3958 | 0.0120 |
| WPLI_Delta_De_N | 0.0655 | 0.3571 | 0.6807 | 0.0002 | 0.1466 | 0.1589 |
| WPLI_Delta_De_R | 0.5171 | 0.0025 | 0.0145 | 0.6694 | 0.1232 | 0.1996 |
| WPLI_Delta_De_T | 0.0190 | 0.6242 | 0.1329 | 0.1815 | 0.0081 | 0.7501 |
| WPLI_Delta_Clo_D | 0.0272 | 0.5570 | 0.2337 | 0.0679 | 0.2196 | 0.0781 |
| WPLI_Delta_Clo_N | 0.0050 | 0.8013 | 0.2223 | 0.0761 | 0.0243 | 0.5793 |
| WPLI_Delta_Clo_R | 0.1338 | 0.1800 | 0.0691 | 0.3438 | 0.1294 | 0.1878 |
| WPLI_Delta_Clo_T | 0.0326 | 0.5197 | 0.1225 | 0.2009 | 0.0527 | 0.4105 |
| WPLI_Delta_CC_D | 0.0000 | 0.9886 | 0.2395 | 0.0641 | 0.1960 | 0.0985 |
| WPLI_Delta_CC_N | 0.0412 | 0.4682 | 0.7448 | 0.0000 | 0.0302 | 0.5357 |
| WPLI_Delta_CC_R | 0.0251 | 0.5725 | 0.0134 | 0.6811 | 0.0466 | 0.4399 |
| WPLI_Delta_CC_T | 0.2510 | 0.0571 | 0.2933 | 0.0370 | 0.0760 | 0.3200 |
| WPLI_Gamma_Bet_D | 0.2615 | 0.0514 | 0.5658 | 0.0012 | 0.0725 | 0.3317 |
| WPLI_Gamma_Bet_N | 0.5414 | 0.0018 | 0.2925 | 0.0374 | 0.0001 | 0.9724 |
| WPLI_Gamma_Bet_R | 0.1864 | 0.1080 | 0.0006 | 0.9288 | 0.0170 | 0.6430 |
| WPLI_Gamma_Bet_T | 0.4422 | 0.0068 | 0.1465 | 0.1591 | 0.1449 | 0.1617 |
| WPLI_Gamma_De_D | 0.5146 | 0.0026 | 0.0050 | 0.8015 | 0.0043 | 0.8158 |
| WPLI_Gamma_De_N | 0.3780 | 0.0147 | 0.1858 | 0.1087 | 0.1719 | 0.1244 |
| WPLI_Gamma_De_R | 0.6818 | 0.0001 | 0.1003 | 0.2502 | 0.0587 | 0.3842 |
| WPLI_Gamma_De_T | 0.2603 | 0.0520 | 0.8518 | 0.0000 | 0.1637 | 0.1347 |
| WPLI_Gamma_Clo_D | 0.5670 | 0.0012 | 0.0002 | 0.9598 | 0.0090 | 0.7371 |
| WPLI_Gamma_Clo_N | 0.4596 | 0.0055 | 0.1042 | 0.2406 | 0.0708 | 0.3379 |
| WPLI_Gamma_Clo_R | 0.1136 | 0.2192 | 0.9417 | 0.0000 | 0.1046 | 0.2397 |
| WPLI_Gamma_Clo_T | 0.3353 | 0.0237 | 0.8707 | 0.0000 | 0.1623 | 0.1366 |
| WPLI_Gamma_CC_D | 0.7575 | 0.0000 | 0.2246 | 0.0743 | 0.0043 | 0.8172 |
| WPLI_Gamma_CC_N | 0.3117 | 0.0305 | 0.0301 | 0.5364 | 0.0629 | 0.3673 |
| WPLI_Gamma_CC_R | 0.2964 | 0.0359 | 0.0113 | 0.7060 | 0.0585 | 0.3852 |
| WPLI_Gamma_CC_T | 0.4962 | 0.0034 | 0.5358 | 0.0019 | 0.2138 | 0.0826 |
| WPLI_Theta_Bet_D | 0.0167 | 0.6464 | 0.0216 | 0.6013 | 0.0063 | 0.7792 |
| WPLI_Theta_Bet_N | 0.2053 | 0.0898 | 0.0018 | 0.8816 | 0.0314 | 0.5273 |
| WPLI_Theta_Bet_R | 0.0005 | 0.9359 | 0.0310 | 0.5299 | 0.0010 | 0.9092 |
| WPLI_Theta_Bet_T | 0.6417 | 0.0003 | 0.0546 | 0.4018 | 0.3141 | 0.0298 |
| WPLI_Theta_De_D | 0.5987 | 0.0007 | 0.2768 | 0.0440 | 0.3386 | 0.0229 |
| WPLI_Theta_De_N | 0.0048 | 0.8054 | 0.0027 | 0.8549 | 0.0000 | 0.9898 |
| WPLI_Theta_De_R | 0.0484 | 0.4308 | 0.0370 | 0.4922 | 0.0014 | 0.8963 |
| WPLI_Theta_De_T | 0.1068 | 0.2345 | 0.0008 | 0.9192 | 0.0160 | 0.6530 |
| WPLI_Theta_Clo_D | 0.5364 | 0.0019 | 0.2100 | 0.0858 | 0.5445 | 0.0017 |
| WPLI_Theta_Clo_N | 0.0246 | 0.5764 | 0.0084 | 0.7451 | 0.0739 | 0.3269 |
| WPLI_Theta_Clo_R | 0.1946 | 0.0998 | 0.5975 | 0.0007 | 0.0067 | 0.7724 |
| WPLI_Theta_Clo_T | 0.2908 | 0.0380 | 0.0197 | 0.6175 | 0.0007 | 0.9241 |
| WPLI_Theta_CC_D | 0.5566 | 0.0014 | 0.0026 | 0.8559 | 0.0488 | 0.4289 |
| WPLI_Theta_CC_N | 0.6195 | 0.0005 | 0.1849 | 0.1096 | 0.1627 | 0.1359 |
| WPLI_Theta_CC_R | 0.5682 | 0.0012 | 0.0024 | 0.8623 | 0.0136 | 0.6794 |
| WPLI_Theta_CC_T | 0.0225 | 0.5939 | 0.5757 | 0.0010 | 0.2891 | 0.0387 |
| WPLI_Band_Bet_D | 0.3089 | 0.0315 | 0.0000 | 0.9893 | 0.0524 | 0.4117 |
| WPLI_Band_Bet_N | 0.0453 | 0.4461 | 0.0909 | 0.2747 | 0.0032 | 0.8413 |
| WPLI_Band_Bet_R | 0.1829 | 0.1118 | 0.0004 | 0.9440 | 0.0381 | 0.4856 |
| WPLI_Band_Bet_T | 0.5319 | 0.0020 | 0.6321 | 0.0004 | 0.0283 | 0.5486 |
| WPLI_Band_De_D | 0.0008 | 0.9227 | 0.0146 | 0.6679 | 0.0311 | 0.5293 |
| WPLI_Band_De_N | 0.1258 | 0.1946 | 0.0462 | 0.4415 | 0.0740 | 0.3265 |
| WPLI_Band_De_R | 0.0321 | 0.5227 | 0.0112 | 0.7074 | 0.0359 | 0.4989 |
| WPLI_Band_De_T | 0.1326 | 0.1822 | 0.2596 | 0.0524 | 0.1252 | 0.1958 |
| WPLI_Band_Clo_D | 0.0257 | 0.5680 | 0.0662 | 0.3547 | 0.0966 | 0.2595 |
| WPLI_Band_Clo_N | 0.0480 | 0.4326 | 0.0181 | 0.6329 | 0.0004 | 0.9463 |
| WPLI_Band_Clo_R | 0.0493 | 0.4262 | 0.0039 | 0.8252 | 0.0311 | 0.5294 |
| WPLI_Band_Clo_T | 0.0517 | 0.4152 | 0.1271 | 0.1922 | 0.0489 | 0.4285 |
| WPLI_Band_CC_D | 0.2381 | 0.0650 | 0.0434 | 0.4564 | 0.0584 | 0.3855 |
| WPLI_Band_CC_N | 0.0821 | 0.3005 | 0.0725 | 0.3318 | 0.1319 | 0.1833 |
| WPLI_Band_CC_r | 0.0343 | 0.5089 | 0.0285 | 0.5473 | 0.0952 | 0.2633 |
| WPLI_Band_CC_T | 0.0134 | 0.6815 | 0.0227 | 0.5923 | 0.0396 | 0.4770 |

WPLI: Weighted Phase Lag Index; De: Degree; CC: Clustering Coefficient; Clo: Closeness Centrality; Bet: Betweenness Centrality; PSD: Power Spectral Density; PE: Permutation Entropy; AE: Approximate Entropy; SAE: Sample Entropy; SPE: Spectral Entropy; LZC: Lempel-Ziv Complexity; Band: Full Frequency Band; T: Pure-tone Paradigm; N: Name-calling Paradigm; R: Reversed-word Sequence Paradigm; D: Different-word Sequence Paradigm

Supplementary Table 5. Classification Results Table of Fusion Features and Combination Features

| **Feature** | **Classifier** | **Accuracy** | **Precision** | **Recall** | **Kappa** | **AUC** |
| --- | --- | --- | --- | --- | --- | --- |
| PC1 | LR | 0.8 | 0.8 | 0.6667 | 0.5714 | 0.8333 |
|  | SVM | 0.8 | 0.8 | 0.6667 | 0.5714 | 0.8519 |
|  | RF | 0.7333 | 0.6667 | 0.6667 | 0.4444 | 0.75 |
|  | KNN | 0.8 | 0.8 | 0.6667 | 0.5714 | 0.8056 |
|  | DT | 0.7333 | 0.6667 | 0.6667 | 0.4444 | 0.7222 |
| PC2 | LR | 0.6 | 0 | 0 | 0 | 0.1667 |
|  | SVM | 0.6 | 0 | 0 | 0 | 0 |
|  | RF | 0.5333 | 0.4 | 0.3333 | 0 | 0.5 |
|  | KNN | 0.5333 | 0 | 0 | -0.129 | 0.2222 |
|  | DT | 0.5333 | 0.4 | 0.3333 | 0 | 0.5 |
| Combined_nonlinear | LR | 0.7333 | 0.6667 | 0.6667 | 0.4444 | 0.7593 |
|  | SVM | 0.7333 | 0.6667 | 0.6667 | 0.4444 | 0.7222 |
|  | RF | 0.8 | 0.8 | 0.6667 | 0.5714 | 0.6852 |
|  | KNN | 0.7333 | 0.6667 | 0.6667 | 0.4444 | 0.6759 |
|  | DT | 0.6667 | 0.6 | 0.5 | 0.2857 | 0.6389 |
| Combined_graph | LR | 0.6 | 0.5 | 0.5 | 0.1667 | 0.7407 |
|  | SVM | 0.7333 | 0.625 | 0.8333 | 0.4737 | 0.6667 |
|  | RF | 0.6 | 0.5 | 0.5 | 0.1667 | 0.7037 |
|  | KNN | 0.7333 | 0.625 | 0.8333 | 0.4737 | 0.713 |
|  | DT | 0.5333 | 0.4 | 0.3333 | 0 | 0.5 |
| Combined_PSD | LR | 0.7333 | 0.6667 | 0.6667 | 0.4444 | 0.7593 |
|  | SVM | 0.8 | 0.7143 | 0.8333 | 0.5946 | 0.6852 |
|  | RF | 0.7333 | 0.6667 | 0.6667 | 0.4444 | 0.8148 |
|  | KNN | 0.8 | 0.8 | 0.6667 | 0.5714 | 0.7222 |
|  | DT | 0.8667 | 0.8333 | 0.8333 | 0.7222 | 0.8611 |
| Combined_all_feature | LR | 0.8667 | 0.8333 | 0.8333 | 0.7222 | 0.7963 |
|  | SVM | 0.7333 | 0.6667 | 0.6667 | 0.4444 | 0.6852 |
|  | RF | 0.8 | 0.7143 | 0.8333 | 0.5946 | 0.7407 |
|  | KNN | 0.8 | 0.8 | 0.6667 | 0.5714 | 0.75 |
|  | DT | 0.8 | 0.7143 | 0.8333 | 0.5946 | 0.8056 |

Combined_nonlinear: All combinations of nonlinear dynamic features selected through statistical testing; Combined_PSD: All power spectrum feature combinations selected through statistical testing will be combined; Combined_graph : Combine all functional connectivity features selected through statistical testing; Combined_all_feature: All combinations of functional features selected through statistical testing; Fused features obtained from principal component analysis (PC1-PC3)

Supplementary Table 6. Regression Results Table of Fusion Features and Combination Features

| Feature | OLS | | SVR | |
| --- | --- | --- | --- | --- |
|  | R^2^_score | P_ score | R^2^_ score | P_ score |
| PC1 | 0.1178 | 0.2105 | 0.2164 | 0.0806 |
| PC2 | 0.0003 | 0.9479 | 0.0235 | 0.5855 |
| Combined_nonlinear | 0.0587 | 0.3844 | 0.3401 | 0.0225 |
| Combined_graph | 0.1640 | 0.1342 | 0.0014 | 0.8956 |
| Combined_PSD | 0.0000 | 0.9945 | 0.2992 | 0.0348 |
| Combined_all_feature | 0.0019 | 0.8762 | 0.4076 | 0.0104 |

Combined_nonlinear: All combinations of nonlinear dynamic features selected through statistical testing; Combined_PSD: All power spectrum feature combinations selected through statistical testing will be combined; Combined_graph : Combine all functional connectivity features selected through statistical testing; Combined_all_feature: All combinations of functional features selected through statistical testing; Fused features obtained from principal component analysis (PC1-PC3)

Supplementary Figure 1. Representative EEG characteristics across the four auditory paradigms (The left column shows a representative valid 19-channel EEG segment after being processed by the preprocessing program. The middle column presents the scalp topographic distribution of EEG activity based on the mean RMS amplitude across valid epochs within the predefined post-stimulus analysis window. The right column shows the corresponding absolute PSD distribution across the predefined frequency bands (delta, theta, alpha, beta, and gamma), calculated from valid epochs.)
